# Estimating prevalence of post-war health disorders using multiple systems data

**DOI:** 10.1038/s41598-024-65478-3

**Published:** 2024-06-26

**Authors:** Prajamitra Bhuyan, Kiranmoy Chatterjee

**Affiliations:** 1 Indian Institute of Management Calcutta, Kolkata, 700104 India; 2 Bidhannagar College, Kolkata, 700064 India

**Keywords:** Amyotrophic lateral sclerosis, Gulf war, Humanitarian crisis, MCEM algorithm, 9/11 terrorist attacks, Health care, Mathematics and computing

## Abstract

Effective surveillance on the long-term public health impact due to war and terrorist attacks remains limited. Such health issues are commonly under-reported, specifically for a large group of individuals. For this purpose, efficient estimation of the size or undercount of the population under the risk of physical and mental health hazards is of utmost necessity. A novel trivariate Bernoulli model is developed allowing heterogeneity among the individuals and dependence between the sources of information, and an estimation methodology using a Monte Carlo-based EM algorithm is proposed. Simulation results show the superiority of the performance of the proposed method over existing competitors and robustness under model mis-specifications. The method is applied to analyse two real case studies on monitoring amyotrophic lateral sclerosis (ALS) cases for the Gulf War veterans and the 9/11 terrorist attack survivors at the World Trade Center, USA. The average annual cumulative incidence rate for ALS disease increases by $33\%$ and $16\%$ for deployed and no-deployed military personnel, respectively, after adjusting the undercount. The number of individuals exposed to the risk of physical and mental health effects due to WTC terrorist attacks increased by $42\%$. These results provide interesting insights that can assist in effective decision-making and policy formulation for monitoring the health status of post-war survivors.

## Introduction

Terrorism has become a serious issue for mankind almost all over the world since the last century. The 1983 Beirut barracks bombings, the 9/11 Twin Towers attacks comprising a series of airline hijackings and four coordinated suicide terrorist attacks in 2001 in the USA, and the 2008 Mumbai Terrorist attacks on 26/11 are some of the horrifying events to mention, but a few in this context. On average, 24,000 people succumbed to terrorist attacks worldwide each year over the last decade^1^. On the other hand, war tends to be more widespread, and its effects are more devastating as it is waged by states with armies equipped with lethal weapons of mass destruction. In recent times, civilian populations are more vulnerable and suffer higher casualties than professional soldiers due to the rise in civil wars and new methods of warfare^2^. Some studies suggest that 200 million people died directly or indirectly as a result of several conflicts during the twentieth century, more than half of them were civilians^3^. Apart from the loss of human lives due to wars and terrorist attacks, a huge number of people suffer from psychological trauma, deteriorating physical and economic conditions, forced migration, injury, disease, and lack of essential amenities like food, water, or energy supplies^4^. Recently experts have also been concerned about the spread of COVID-19 and mental health consequences due to the Russia–Ukraine conflict^5^. Apart from civilians, a significant number of soldiers suffer from post-traumatic stress disorder on returning from war zones^3^. In the World Health Assembly^6^, the WHO reported that $10\%$ of the people, who experience traumatic events due to armed conflicts, will have serious mental health problems, and another 10% will develop behaviour that will hinder their ability to function effectively.

The amount of losses, in terms of deaths, physical injuries, and psychological disorders caused by terrorist activity or war, are closely monitored by federal agencies. This helps to identify the actual victims who encountered such losses and formulate policies for the welfare of the survivors and the families of the deceased. However, death registration systems in most of the under-developed countries are not satisfactory, which might result in a considerable amount of under-ascertainment of deaths and injuries in several terrorist activities and wars^7^. Even in the case of a developed country like USA, the vast and varied consequences of wars and counter-terror operations, since the deadliest 9/11 incident, were not properly accounted for^8^. The UN Human Rights Council reported nearly 350,209 identified deaths since 2014 during the long-lost Syria conflict from March 2011 to March 2021. Later, some leading UK-based monitoring groups indicated a notable amount of undercount in the number of deaths provided by the UN Human Rights Council^9^. In this article, the extent of under-ascertainment in estimating the number of victims suffering from a war or terrorist activity is examined. First, we discuss two case studies on the prevalence of (1) amyotrophic lateral sclerosis (ALS) disease among the US military personnel during the 1991 Gulf War, and (2) physical and mental disorders post WTC Twin Tower terrorist attacks on September 11, 2001, and related inferential issues in the next section. Then we propose a generic model that encompasses the time variation, list-dependence and individual heterogeneity. A novel Monte Carlo-based expectation-maximization algorithm for the estimation of the model parameters is proposed. To compare the performance of our proposed method with that of the existing competitors and for sensitivity analyses concerning model assumptions, an extensive simulation study has been conducted. Next, analyses of real datasets on ALS surveillance due to the Gulf War and Twin Towers population are presented based on our model as well as the relevant competitors. Finally, we conclude with some discussion on future research directions.

## Multiple systems data and related issues

The Middle East is one of the most affected regions due to war in the last couple of decades^1^. The Iran–Iraq war (1980–1988), the Iraqi invasion of Kuwait resulting in the Gulf War (1991), and the US-led attack on Iraq in 2003 had a profound impact on the health of civilians and army personnel. Around $79\%$ of US military troops reported at least one chronic medical condition^10^. We consider the nationwide epidemiological investigation of ALS among the US military veterans in the Gulf War^11^. ALS is a rare neurological disease that primarily affects the nerve cells responsible for controlling voluntary muscle movements like chewing, walking, and talking. The disease is progressive, and currently, there is no cure for ALS. Due to the rarity of ALS, under-ascertainment could have a substantive effect on the rates and associated risk estimate^12^. Hence, estimating total ALS cases due to the 1991 Gulf War is important to assess the spread of the disease among US military personnel.

**Figure 1:**
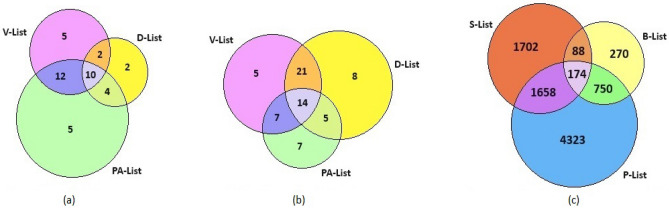
Counts and overlaps of the three available data lists for each of the (**a**) deployed, (**b**) non-deployed Gulf War veterans; and (**c**) victims in the WTC attack on 9/11.

We consider the multiple systems estimation (MSE) approach for estimating the prevalence of ALS disease in the context of the aforementioned problem. This approach collects information on the bona fide US military personnel having ALS from both passive and active ascertainment activities^11^. Passive methods involve self-registration via a toll-free telephone number, notices published on relevant internet sites, and mass mailings of study brochures to practicing neurologists affiliated with the American Academy of Neurology and to members of Veteran Service Organizations. Participants were also identified from a survey conducted by the ALS Association among its members. Further, active screening of the Veterans Association benefit files, Department of Defense inpatient and outpatient records, pharmacy medical databases, National Death Index files, and military insurance policy dossier was conducted to improve coverage. From these aforementioned sources, four lists of ALS-affected Gulf War veterans are prepared: (1) Veterans Affairs database (V-List), (2) Department of Defense database (D-List), (3) Phone-line database (P-List), and (4) ALS Association database (A-List). In total, 107 verified ALS cases were reported in the troops during the 10 years beginning from August 2, 1990, and 40 out of these cases were from 696,118 deployed, and the remaining 67 were from 1,786,215 non-deployed military personnel. In the present study, we merge the data from the P and A lists and rename it as ‘PA-List’ to avoid sparsity in the data from both the P and A lists. By matching the identities from the V, D, and PA lists, overlap counts between the lists are presented through Venn diagrams for each of the deployed and non-deployed veterans (see Fig. 1a,b). This dataset is typically called a triple record system (TRS) which is equivalent to a capture-recapture data with three capture attempts. To assess the prevalence of the ALS disease among military personnel, an estimate of the total number of ALS cases is required. This problem is equivalent to the estimation of the count of ALS cases among the veterans who are not included in any of the three aforementioned lists.

Another study of our present interest is associated with one of the deadliest terrorist attacks ever carried out on US soil, when four aeroplanes were hijacked and two of them were flown into the Twin Towers of the World Trade Center (WTC) in New York City on September 11, 2001. In addition to the loss of lives, the survivors were exposed to hazardous dust and debris from the collapsed buildings and many people suffered from psychological trauma. The exact number of individuals present in the Twin Towers of the WTC at 8:46 AM on the day of the attack is not exactly known. To study the long- and short-term physical and mental health effects of the survivors, researchers attempted to trace eligible individuals from various sources, including the World Trade Center Health Registry (WTCHR)^13^. It helps to determine the extent of persistent physical and mental health issues and identify if any new symptoms and conditions have emerged. Individuals who received information from their employers, community outreach, or media campaigns could register by calling a toll-free number or through WTCHR website. A sample list purchased from Genesys Sampling Systems and enlisted businesses were contacted for WTCHR. Two additional lists of organizations were also obtained at no cost to improve the coverage. In May 2004, the Port Authority provided a list of individuals who were issued a security badge to one or more of the seven WTC buildings during the five years after the attack. Due to budget and time constraints, enlisted individuals only with complete information were traced. Finally, a multiple systems database comprising three lists is prepared^14,15^: (1) self-identified individuals list (S), (2) businesses list (B), and (3) port authority list from New York and New Jersey Ports (P). Similar to the ALS cases, TRS data on WTC Twin Tower survivors are presented in Fig. 1c. However, the available information does not readily provide the actual number of individuals exposed to the risk of post-attack trauma. The under-reporting of the survivors causes a sizable bias in evaluating the severity and aftereffects of such horrific incidents. Therefore, in this article, we primarily confined ourselves to estimating the size of the survivors of the 9/11 WTC attack.

In both the aforementioned studies, the group of victims remained fixed and unaltered, which satisfies the basic assumption of *closed population* for MSE. The inclusion status of an individual in any list may have a direct causal effect on his/her inclusion in other lists. This phenomenon is known as *list-dependence* caused by behavioural response variations^16,17^. Both the case studies under consideration are aimed at formulating healthcare policies for the welfare of the respective victims in several means. In a capture-recapture study for economic or healthcare welfare, eligible members of the population are more prone to include themselves in the subsequent attempts after their first inclusion compared to those who have not been enlisted before^18^. Moreover, several researchers advocated that recapture proneness (i.e. positive dependence due to the behavioural response variation) is commonly encountered in studies related to epidemiology and healthcare surveillance^19,20^. Also, the lack of homogeneity in capture probabilities over the individuals induces positive dependence among the lists. Note that the presence of *list-dependence* causes *correlation bias* in the estimate of the population size obtained by assuming independence among the lists^21,22^. Therefore, modeling TRS data becomes more challenging if *individual heterogeneity* is present along with list-dependence caused by behavioural response variation. In particular, the US military personnel in the Gulf War were more ethnically diverse than their counterparts deployed in previous wars^23^. This phenomenon potentially induces a non-negligible amount of heterogeneity in the inclusion probabilities of veterans in different lists available for reporting ALS disease. For similar reasons, heterogeneity in the inclusion probabilities exists in the TRS datasets related to WTC terrorist attack^15^. We validated this using a graphical test using *Rcapture* package in R software^24^. For details, see Section B of the Supplementary Material. We also checked the existence of *list-dependence* between the three lists in both the TRS datasets through a classification technique proposed by Chatterjee and Mukherjee^25^ and found that the lists in both datasets are positively correlated.

In this context, the ecological model $M_{tb}$ accounts for the list dependence in terms of the behavioural response variation. Chatterjee and Bhuyan^22^ pointed out several limitations of the model $M_{tb}$ for analysing TRS data. Log-linear models are also popularly used for the same purpose^26,27^, but the associated parameters are not well interpretable to the practitioners^28^. On the other hand, quasi-symmetric or partial quasi-symmetric Rasch models can be used to estimate the population size in the presence of *individual heterogeneity*^29^. For the most general scenario, Bartoucci and Pennoni^30^ proposed a latent Markov model for capture-recapture data encompassing *time variation*, *individual heterogeneity* and *list-dependence*. In this context, the nonparametric sample coverage approach has been proposed by Chao and Tsay^31^. However, it may provide infeasible estimates in many instances. See Section E of the Supplementary Material for more details on the relevant existing models and estimators. In this article, we propose a novel model and an efficient algorithm for estimation of the size of a population that deals with both the *list-dependence* and *individual heterogeneity*. Interestingly, the parameters associated with the proposed model possess nice interpretations and have a much wider domain of applicability in the fields of public health, demography, and social sciences.

## Model and methods

### Modeling TRS data

In a capture-recapture setup, it is evident that the available lists are potentially dependent due to behavioural response variations for some or all of the individuals in the population of interest. Here, we propose a model to account for such inherent dependence among the lists in a TRS. Let us denote $\left( Z_h^{(1)}, Z_h^{(2)},Z_h^{(3)}\right)$ as the actual inclusion status of the *h*th individual in the three lists $L_1$, $L_2$, and $L_3$, for $h=1,2,\ldots ,N$, where *N* denotes the unknown size of the population. The capture status $Z_h^{(s)}$ takes the value 1 or 0, denoting the presence or absence of the *h*th individual in the *s*-th list, for $s=1,2,3$ and we can formally write the model for inclusion status of individuals to account the *list-dependence* among the three lists and *individual heterogeneity* as:

1 $$\begin{aligned} \left( Z_h^{(1)},Z_h^{(2)}, Z_h^{(3)}\right) = {\left\{ \begin{array}{ll} \left( X_{1h},X_{1h},X_{3h}\right) &{} \text{ with } \text{ probability } \alpha _{1},\\ \left( X_{1h},X_{2h},X_{2h}\right) &{} \text{ with } \text{ probability } \alpha _{2},\\ \left( X_{1h},X_{2h},X_{1h}\right) &{} \text{ with } \text{ probability } \alpha _{3},\\ \left( X_{1h},X_{1h},X_{1h}\right) &{} \text{ with } \text{ probability } \alpha _{4},\\ \left( X_{1h},X_{2h},X_{3h}\right) &{} \text{ with } \text{ probability } \left( 1-\alpha _0\right) , \end{array}\right. } \end{aligned}$$

where $\alpha _0=\sum _{s=1}^{4}\alpha _{s}$ and $X_{sh}$’s are independent Bernoulli random variables with parameters $\mathcal {P}_{sh}$ for all $h=1,\ldots ,N$, and $s=1,2,3$. To incorporate *individual heterogeneity*, here we consider $\mathcal {P}_{sh}$’s to be realizations of an independent and identically distributed random variable with support (0, 1), for each $s=1,2,3$. Further, it is noted that the attempts to capture an individual by the three available sources in each of the two case studies are not time-ordered^11,15^. However, our proposed model (1) can accommodate both the *time-ordered* and *time-unordered* situations in a very novel fashion. For instance, the first-order pairwise positive dependence between lists ($L_1$ and $L_2$) is modeled as $X_{2h}=X_{1h}$ for $\alpha _{1}$ proportion of individuals. It means that for each of all the $\alpha _{1}$ proportion of individuals, inclusion statuses in List 1 and List 2 are identical. Similarly, inclusion status in List 3 is the same as that of List 2 (List 1) for $\alpha _{2}$ ($\alpha _{3}$) proportion of individuals and $\alpha _{4}$ proportion of individuals represents joint interaction among the three lists. Therefore, the remaining $(1-\alpha _0)$ proportion of individuals, behave independently over the three lists. We refer to this generic model as the Trivariate Heterogeneous Bernoulli model (THBM) as it incorporates *individual heterogeneity* and *behavioural dependence* arising from different lists. Note that the primary objective is to estimate the population size *N*. In the next subsection, we propose an estimation methodology of the model parameters based on the MCEM algorithm, providing a generic solution to the identifiability issue. See Section C.1. in Supplementary Material for identifiability issues.

### Estimation methodology

**Figure 2:**
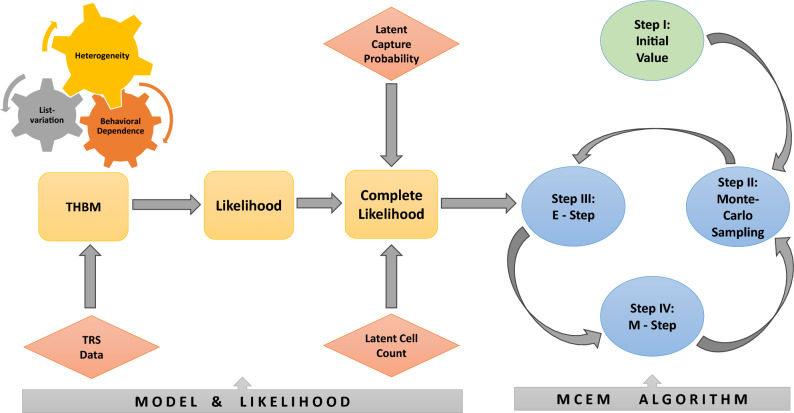
Diagrammatic presentation of the estimation methodology.

The vector of observed counts in TRS for each of the two datasets (presented in Fig. 1) can be presented in an incomplete contingency table where one cell is structurally unobserved (see Section A in the Supplementary Material). Therefore, in the context of MSE, population size is traditionally estimated based on the likelihood theory, where the vector of observed counts, follows a multinomial distribution with index parameter *N*. However, the likelihood function is complicated in the presence of the first and second-order *list-dependence*, along with *individual heterogeneity* in terms of the variations in capture probabilities. The estimation of *N* and other associated parameters in (1) is computationally challenging due to the involvement of intractable numerical integrals^32^. We propose a novel method of estimation based on *Monte Carlo expectation-maximization* (MCEM) algorithm adopting a data augmentation strategy to simplify the likelihood function. As a result, the complete data likelihood possesses a simple form as a product of the power functions of the parameters. Then, we employ a similar approach as suggested by Carle and Strub^33^ to obtain a likelihood function free of the nuisance parameters. To implement the MCEM algorithm, we generate samples from the conditional distributions of augmented data given the observed data and parameters. We employ an iterative algorithm close in spirit to the method of *EM-within-Gibbs* and stochastic *EM-within-Gibbs* considered by Chatterjee and Mukherjee^34^. The estimation methodology is diagrammatically presented in Fig. 2. See Section C.2 of the Supplementary Material for more details.

## Results

### Simulation study

The performance and robustness of the proposed estimator are evaluated through extensive simulation experiments. We consider eight different compositions of populations representing varying degrees of *list-dependence* and *individual heterogeneity* in capture probabilities (see Table F.1 in the Supplementary Material). We generate 1000 datasets from the proposed model keeping the total population size *N* fixed at 500 and 1000 for each of these eight different choices of populations, and compute the relative mean absolute error (RMAE). We obtain $95\%$ interval (C.I.) for *N* based on the log-transformation method, proposed by Chao^35^. Following Chatterjee and Mukherjee^36^, we compute the length of the $95\%$ confidence interval (LCI) as well as its coverage probability (CP). To evaluate and compare the performance of the proposed estimator with the existing competitors (see Section E of the Supplementary Material for details), we consider log-linear model (LLM)^26^, $M_{tb}$ model^22,37^, quasi-symmetry model (QSM)^16^, partial quasi-symmetry model (PQSM)^16^, non-parametric sample coverage method (SC)^31^ and the independent model (IM) i.e. LLM without interaction effects^26^. See Section F of the Supplementary Material for a detailed comparative analysis of the performances of different methods. The results found that the proposed model performs the best in terms of RMAE over all the populations for both $N=500$ and 1000. Moreover, the CPs of the proposed THBM estimator are higher than all the existing competitors.

To study the effect of model misspecification when the data-generating mechanism deviates from the fitted model, we analyse the sensitivity of the estimates based on the proposed THBM in comparison with the existing estimators. For this purpose, we generate data using a mechanism that induces an additional dependence among the capture statuses similar to a first-order auto-regressive model as considered in Chao^35^. We also generate data from the Rasch model^29^ for the same purpose. In both cases, the proposed estimator outperforms the existing competitors with respect to RMAE. Overall, the CPs of the proposed estimator are close to $100\%$ and much better than all other estimators. See Section F.1 of the Supplementary Material.

### Applications

#### ALS monitouring data for Gulf War veterans

First, we consider the case of ALS disease among the 1991 Gulf War veteran military personnel. As per the report of the Committee on Gulf War and Health^23^, nearly 697000 US troops were developed over the course of buildup and the war from August 1990–February 1991. Besides injuries and deaths among the coalition forces, a large number of veterans suffered from various health-related issues during and after the war, which persisted for more than 25 years. From studies conducted since 1995, it has been found that the deployed Gulf War veterans suffered more than their non-deployed counterparts, both in numbers and severity^23^. In particular, $9.4\%$ of deployed veterans suffered from a gross neurological disorder, whereas the figure is $6.3\%$ for non-deployed personnel. There is a matter of serious concern among the researchers regarding possible under-ascertainment of the cases^11^. Since ALS is a rare disease, under-ascertainment could have a substantive effect on the rates and associated risks^11^. Therefore, in this report, our prime interest is to estimate the size or under-ascertainment of the deployed and non-deployed veterans affected by ALS based on the data provided in Fig. 1.

**Figure 3:**
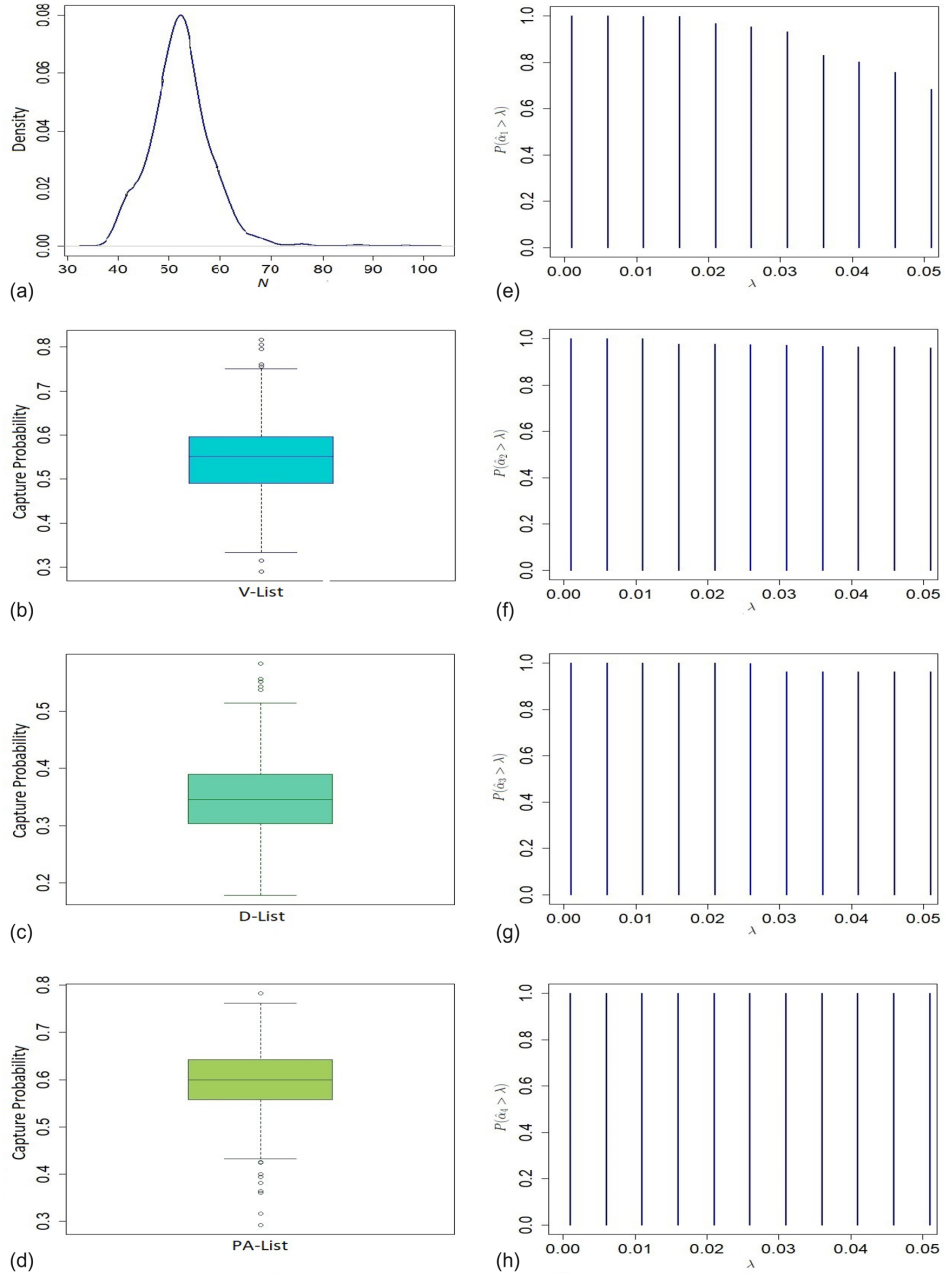
(**a**) Presents density of the size of the population of deployed veterans in the Gulf War; (**b**)–(**d**) present boxplots of capture probabilities associated with V-List, D-List, and PA-List respectively; (**e**)–(**h**) present tests of significance of four dependence model parameters $\alpha _1, \alpha _2, \alpha _3$ and $\alpha _4$, respectively.

**Figure 4:**
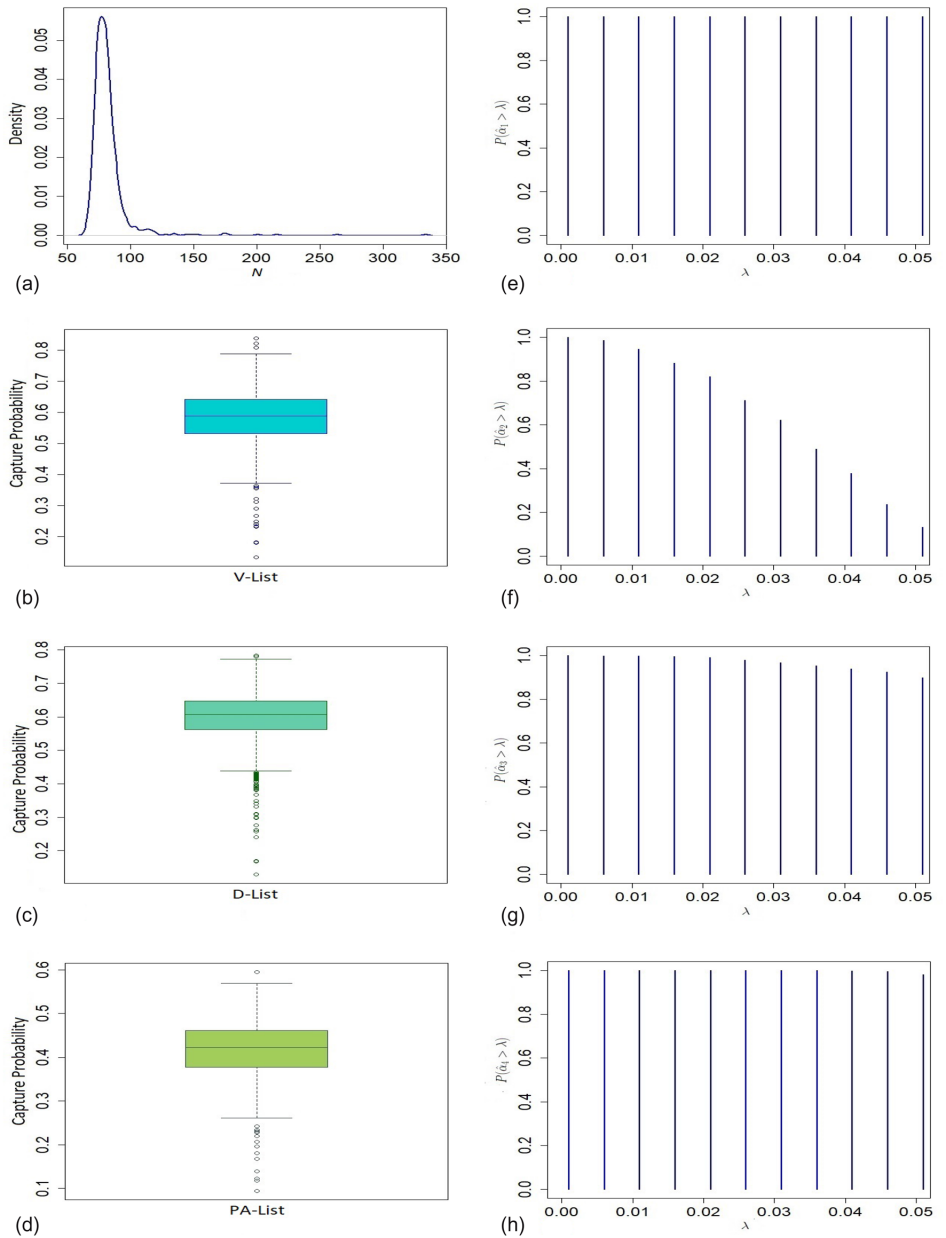
(**a**) Presents density of the size of the population of non-deployed veterans in the Gulf War; (**b**)–(**d**) present boxplots of capture probabilities associated with V-List, D-List, and PA-List respectively; (**e**)–(**h**) present tests of significance of four dependence model parameters $\alpha _1, \alpha _2, \alpha _3$ and $\alpha _4$, respectively.

We compute the estimate of the prevalence of ALS-affected deployed and non-deployed veterans and corresponding $95\%$ confidence intervals based on 1000 nonparametric bootstrap samples. In the capture-recapture setting, point estimators of population size commonly possess positively skewed distributions. Therefore, the confidence interval for the prevalence is computed based on the log-transformation method proposed by Chao^35^. See Table G.2 in the Supplementary Material for details. Using a computer equipped with 32 GB RAM and a 2.59 GHz Intel Core(TM) i7-8850H processor, analysing a bootstrap sample only takes 25 s on average. The estimate of the ALS-affected veterans, based on THBM, is greater than that of all other methods under consideration. All the competitors, except model $M_{tb}$, yield similar estimates of the number of ALS cases for both the deployed and non-deployed veterans and indicate a relatively low (moderate) rate of undercount (rate of undercount is defined as $(\hat{N}-n)/\hat{N}$, where $\hat{N}$ and *n* denote estimate of *N*, and sum of the observed cell counts in a TRS, respectively) for deployed (non-deployed) personnel. Surprisingly, no undercount of ALS cases is observed for the deployed veterans based on model $M_{tb}$ in contrast to the findings of all other methods. The $95\%$ confidence intervals based on PQSM and LLM are extremely wide for both the deployed and non-deployed cases, respectively. Lum et al.^38^ argued that large levels of uncertainty may imply drastic violations of assumptions and hence produce such unrealistic findings.

The estimated undercount of ALS cases for the deployed veterans ($33\%$) is significantly higher than that of the non-deployed ($16\%$) personnel based on the proposed model. In total, 131 ALS cases are estimated based on THBM with $95\%$ confidence interval (110, 193) for aggregated over deployed and non-deployed veterans. Figures 3a and 4a show long-tail density plots based on the bootstrap estimates of the sizes of deployed and non-deployed veterans in the war, respectively. For testing significance of the dependence parameters $\alpha _{i}$’s, we plot $P\left[ \hat{\alpha }_{i}> \lambda \right]$ against different values of significance level $\lambda$ based on the bootstrap samples for $i=1,\ldots ,4$ (See Figs. 3e–h, 4e–h). The bulk of the sampling distribution for almost all the dependence parameters for both deployed and non-deployed cases are away from 0 implying their significance. Their estimates indicate that the three lists are dependent on each other for $50\%$ ($41\%$) of the deployed (non-deployed) veterans. For analysing the completeness of different lists, we presented boxplots of marginal capture probabilities for both deployed and non-deployed cases in Figs. 3b–d and 4b–d, respectively. The completeness of the V list for both the deployed and non-deployed veterans is similar (55% for deployed and 60% for non-deployed cases). Like the V list, non-deployed cases are enlisted at a higher rate (62%) in the D list compared to their deployed counterparts (34%). In contrast, ALS cases among the deployed (59%) are more prevalent than those among the non-deployed personnel (42%) in the combined list PA.

The average annual cumulative incidence rate (AACIR) (AACIR=$\frac{\text {Number of cases of a disease}}{\text {Number of years} \times \text {Number of persons at risk}}\times 100000$

) of ALS among deployed and non-deployed military personnel were computed as 0.57 and 0.38 per 100,000 persons, respectively^11^. However, these figures did not account for the underreported cases, and any conclusion drawn from these statistics may be misleading. In particular, the relative risk of post-war ALS is underestimated when underreporting is higher among the Gulf War deployed compared to that of the non-deployed personnel. Therefore, it is essential to estimate the underreported cases and adjust the calculation of the AACI for an unbiased evaluation of the post-war syndromes. As per our estimate, we found that the AACIR of ALS among Gulf War veterans increases significantly to 0.76 per 100,000 persons, and the same for non-deployed personnel increases marginally to 0.44 per 100,000 persons. After adjusting the undercount, the AACIR for ALS disease increases by $33\%$ and $16\%$ for deployed and no-deployed military personnel, respectively. In other words, the relative risk of ALS cases increases by almost $15\%$.

#### WTC survivors data

Several reports by the Public Health Care Response organizations established a direct physical and mental impact on the survivors^39^. Unfortunately, no official records have been kept on the casualties of the whole 9/11 attacks either by the FBI or the New York City administration in their respective crime records^40^. As a result, surveillance on the health condition of survivors has not been done systematically over time. Thus, it is an absolute necessity to gain an idea about the actual count of the survivors to make effective policy decisions to trace unidentified individuals for their well-being of physical and mental health^41^.

**Figure 5:**
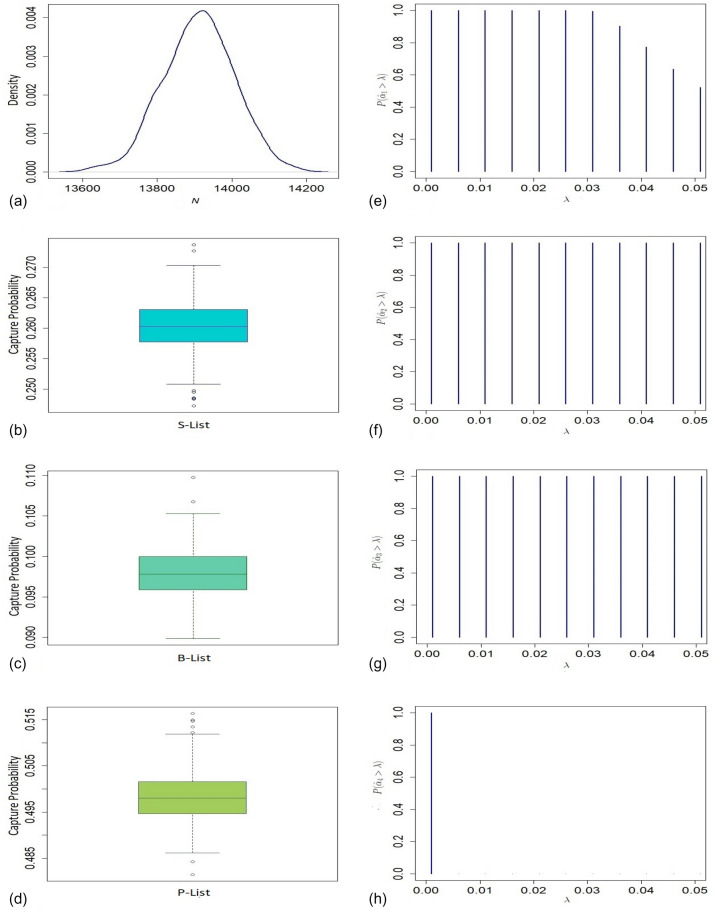
(**a**) Presents density of the size of the population at the time of the attack in WTC Twin Towers on 9/11; (**b**)–(**d**) present boxplots of the capture probabilities associated with the S-List, B-List, and P-List, respectively; (**e**)–(**h**) present tests of significance of four dependence model parameters $\alpha _1, \alpha _2, \alpha _3$ and $\alpha _4$, respectively.

As per the proposed estimation method, 13919 individuals were present in the Twin Towers when the first plane hit the north tower with $95\%$ confidence interval (13733, 14112). The bootstrap distribution of the population size is presented in Fig. 5a which shows a symmetric distribution of the estimates. Here also, the estimated undercount based on model $M_{tb}$ is unexpectedly low. The $95\%$ confidence intervals for all other competitive estimators are very wide compared to the proposed method (see Table G.2 in Section G of the Supplementary Material). From Fig. 5b–d, we can see that the completeness of S-list, B-list, and P-list are approximately 26%, 10%, and 50%, respectively. The number of registered deaths due to WTC attacks is 2512, which leads to 11767 survivors based on the proposed method^42^. After adjusting the undercount, the number of survivors increased by $42\%$, and all of them were exposed to the risk of short-term and/or long-term physical and mental health effects^13^. In Fig. 5e–h, the bulk of the distribution of all the estimated dependence parameters except that of $\alpha _{4}$ is away from 0, which indicates their significance. This implies that behavioural response variation is adequately incorporated through pairwise list-dependence. Our estimate suggests that the $35.6\%$ of individuals exhibit behavioural dependence among the three secures.

## Concluding remarks

A novel method has been proposed to asses the prevalence of health disorders of the Gulf War survivors and victims of 9/11 terrorist attack on WTC, USA. The proposed method seems to have an edge in terms of flexibility in modeling both the *list-dependence* and heterogeneous catchability. It provides a clear picture of the nature of heterogeneity in capture probabilities and the extent of behavioural dependence among individuals. In contrast to the existing methodology, the proposed method handles the identifiability issues without any implausible restrictions on parameters.

In the context of disease monitoring, we often emphasize the necessity to correct the conventional method of calculating incidence rate adjusting the estimated size of the under-reported cases for an unbiased evaluation of post-war syndromes. Based on our analyses, we found that the average annual cumulative incidence of ALS increases significantly among the Gulf War veterans after adjusting the undercount events. In the context of the WTC terrorist attack, several reports indicated that the survivors, including rescuers, police personnel, and other government officials, are more or less affected by mental and environmental health issues^39,41^. Some of them died after the incident, and the rest carried their losses in their body or mind forever. However, there is not much work available on the risk estimate of post-attack diseases with suitable adjustments for the undercount. The lack of demographic information for this case study hampered our ability to further analyse the risk factors.

The scope of the proposed model goes far beyond the particular case studies under consideration and has much wider applicability. In the last couple of years, multiple systems estimation strategies have been applied to problems related to humanitarian crises, e.g. estimating the number of victims of human trafficking and modern slavery^43^. Recently, due to the COVID-19 pandemic and the Russia-Ukraine conflict, policymakers have been interested in the figures related to the influx of refugees, and the adverse effects of COVID-19 vaccine. In such applications, sparse or even no overlapping lists pose challenges in model fitting^44^. It is an interesting research problem to extend the proposed approach to model such datasets to address the existing estimation issues. Another possible research direction could be allowing heterogeneity among individuals using observable or latent covariates to model the capture probabilities associated with the proposed model^30,45^.

### Supplementary Information

Supplementary Information.

## Data Availability

All data analysed in the current study are included in this article.
