# Supplementary material for: Estimating prevalence of post-war health disorders using multiple systems data
Source: Sci Rep. 2024 Jun 26;14:14763. doi: 10.1038/s41598-024-65478-3 (PMC11208173; doi:10.1038/s41598-024-65478-3)
Supplement: Supplementary file 1 — Supplementary Information. [file 41598_2024_65478_MOESM1_ESM.pdf]

Supplementary Material for "Estimating Prevalence of Post-war Health Disorders using Multiple Systems Data" by Bhuyan and Chatterjee

A TRS Data Structure

Any capture-recapture type data structure generated by a small number of capture attempts is conventionally written in an incomplete contingency table for a closed population. Therefore, capture-recapture type data with three lists (i.e. TRS) can be written in the following incomplete 2^3 contingency table, where the last cell x_{000} remains structurally unobserved.

Table 1: Data structure associated with a triple record system (TRS).

	List 3					
	In			Out		
	List 2			List 2		
List 1	In	Out	Total	In	Out	Total
In	x_{111}	x_{101}	$x_{1\cdot 1}$	x_{110}	x_{100}	$x_{1\cdot 0}$
Out	x_{011}	x_{001}	$x_{0\cdot 1}$	x_{010}	x_{000}	$x_{0\cdot 0}$
Total	$x_{\cdot 11}$	$x_{\cdot 01}$	$x_{\cdot\cdot 1}$	$x_{\cdot 10}$	$x_{\cdot 00}$	$x_{\cdot\cdot 0}$

Note that in the context of multiple systems estimation (MSE), the population size $N = \sum_{i,j,k=0,1} x_{ijk}$ is to be estimated since the cell x_{000} is always unobserved in Table 1. Therefore, in an MSE setup, estimation of N is equivalent to the estimation of $x_{000} = N - n$, where n refers to the sum of all seven observed cells in (1), i.e. $n = x_{111} + x_{110} + x_{101} + x_{011} + x_{100} + x_{010} + x_{001}$. Therefore, in terms of the TRS data structure given in Table 1, we present our three TRS datasets (see Figure 1 in the main article) in the following table.

Table 2: TRS Datasets on ALS disease among the Gulf war veterans and WTC Twin Tower population at the time of 9/11 terrorist attack.

Dataset	Stratum	x_{111}	x_{110}	x_{101}	x_{011}	x_{100}	x_{010}	x_{001}	Total (n)
ALS	Deployed	10	2	12	4	5	2	5	40
	Non-deployed	14	21	7	5	5	8	7	67
WTC		174	88	1658	750	1702	270	4323	8965

B Test for Heterogeneity

In order to validate the assumption of the existence of *individual heterogeneity* in both the datasets, we plot $log\left(f_i / \binom{3}{i}\right)$ against i, using the *plot.descriptive* function in the *Rcapture* package in R, for $i = 1, 2, 3$. Here, f_i denotes the number of individuals captured i times. For further details, see Baillargeon and Rivest (2007). In terms of our notation, $f_1 = x_{100} + x_{010} + x_{001}, f_2 = x_{110} + x_{101} + x_{011}$ and $f_3 = x_{111}$. It is quite clear that the graphs indicate the presence of *individual heterogeneity* for non-deployed veterans and WTC data, whereas a marginal *individual heterogeneity* is supposed to be exist in deployed veterans data (see Figure B.1).

C Estimation Methodology

Traditionally in the context of MSE, population size N is estimated based on the likelihood theory, where the vector of observed cell counts

$$\boldsymbol{x} = \left\{ x_{ijk} : x_{ijk} = \sum_{h=1}^{n} \mathbb{I}\left[Z_h^{(1)} = i, Z_h^{(2)} = j, Z_h^{(3)} = k \right]; i, j, k = 0, 1; i = j = k \neq 0 \right\},$$

as presented in Table 1, follow a multinomial distribution with index parameter N and the associated cell probabilities $\boldsymbol{p} = \{p_{ijk} : i, j, k = 0, 1; i = j = k \neq 0\}$ (Sanathanan, 1972), where $\mathbb{I}[\cdot]$ denotes indicator function and $Z_h^{(1)}$, $Z_h^{(1)}$, $Z_h^{(1)}$ are defined in model (1) in the main article. Therefore, the likelihood function is given by

$$L(N, \boldsymbol{p}|\boldsymbol{x}) = \frac{N!}{\prod_{i,j,k=0,1; i=j=k\neq 0} x_{ijk}!(N - x_0)!} \prod_{i,j,k=0,1} p_{ijk}^{x_{ijk}},$$

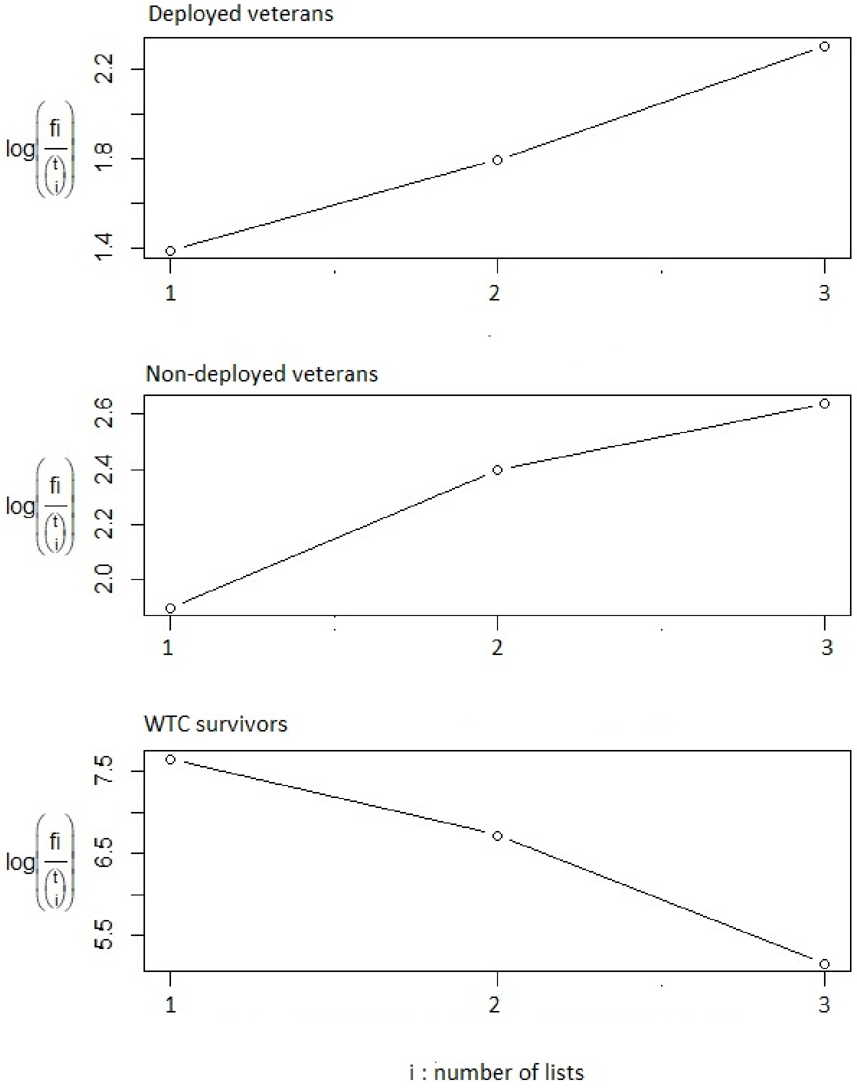

Figure B.1: Graphs showing the trajectory of $log\left(f_i\,/\,\binom{3}{i}\right)$ against i for the deployed, non-deployed veterans data and the WTC data

where $x_{000} = N - n$. We rewrite the likelihood function based on the model (1), presented in the main text as

$$\begin{aligned}
L(N, \boldsymbol{\alpha}, \boldsymbol{\mathcal{P}}|\boldsymbol{x}) \propto\ & \frac{N!}{(N-x_0)!}[(1-\alpha_0)\mathcal{P}_1\mathcal{P}_2\mathcal{P}_3 + \alpha_1\mathcal{P}_1\mathcal{P}_3 + \alpha_2\mathcal{P}_1\mathcal{P}_2 + \alpha_3\mathcal{P}_1\mathcal{P}_2 + \alpha_4\mathcal{P}_1]^{x_{111}} \\
& \times[(1-\alpha_0)\mathcal{P}_1\mathcal{P}_2(1-\mathcal{P}_3) + \alpha_1\mathcal{P}_1(1-\mathcal{P}_3)]^{x_{110}} \\
& \times[(1-\alpha_0)(1-\mathcal{P}_1)\mathcal{P}_2\mathcal{P}_3 + \alpha_2(1-\mathcal{P}_1)\mathcal{P}_2]^{x_{011}} \\
& \times[(1-\alpha_0)\mathcal{P}_1(1-\mathcal{P}_2)(1-\mathcal{P}_3) + \alpha_2\mathcal{P}_1(1-\mathcal{P}_2)]^{x_{100}} \\
& \times[(1-\alpha_0)\mathcal{P}_1(1-\mathcal{P}_2)\mathcal{P}_3 + \alpha_3\mathcal{P}_1(1-\mathcal{P}_2)]^{x_{101}} \\
& \times[(1-\alpha_0)(1-\mathcal{P}_1)\mathcal{P}_2(1-\mathcal{P}_3) + \alpha_3(1-\mathcal{P}_1)\mathcal{P}_2]^{x_{010}} \\
& \times[(1-\alpha_0)(1-\mathcal{P}_1)(1-\mathcal{P}_2)\mathcal{P}_3 + \alpha_1(1-\mathcal{P}_1)\mathcal{P}_3]^{x_{001}} \\
& \times[(1-\alpha_0)(1-\mathcal{P}_1)(1-\mathcal{P}_2)(1-\mathcal{P}_3) + \alpha_1(1-\mathcal{P}_1)(1-\mathcal{P}_3) \\
& +\alpha_2(1-\mathcal{P}_1)(1-\mathcal{P}_2) + \alpha_3(1-\mathcal{P}_1)(1-\mathcal{P}_2) + \alpha_4(1-\mathcal{P}_1)]^{N-x_0}, \quad \text{(C.0.1)}
\end{aligned}$$

where $x_{000} = N - x_0$, $p_{000} = 1 - \sum_{i,j,k=0,1;i=j=k\neq 0} p_{ijk}$, $\boldsymbol{\alpha} = (\alpha_1, \alpha_2, \alpha_3, \alpha_4)$, and $\boldsymbol{\mathcal{P}} = (\mathcal{P}_1, \mathcal{P}_2, \mathcal{P}_3)$. In addition to the first and second order *list-dependence* involved in TBM, the proposed THBM accounts for the *individual heterogeneity* in terms of the variations in capture probabilities considering $\mathcal{P}_s$ as a random effect for $s = 1, 2, 3$. Now, integrating the likelihood function, given in (C.0.1), with respect to the distribution of $\mathcal{P}_l$, we obtain the marginal likelihood function of $\boldsymbol{\theta}$ as

$$L(\boldsymbol{\theta}|\boldsymbol{x}) = \int_{\mathbb{R}^3} L(N, \boldsymbol{\alpha}, \boldsymbol{\mathcal{P}}|\boldsymbol{x}) \times \left\{ \prod_{s=1}^{3} g_{\mathcal{P}_l}(\mathcal{P}_l|\delta_l) \right\} d\mathcal{P}_1 d\mathcal{P}_2 d\mathcal{P}_3, \quad \text{(C.0.2)}$$

where $g_{\mathcal{P}_s}(\cdot|\delta_s)$ is the density of $\mathcal{P}_s$ with parameter δ_s for $s = 1, 2, 3$, and $\boldsymbol{\theta} = (N, \boldsymbol{\alpha}, \boldsymbol{\delta})$. The estimation of N and other associated parameters in THBM is computationally challenging in a conventional frequentist setup due to the involvement of intractable numerical integrals (Coull and Agresti, 1999). One can consider approximation methods for numerical integration like Monte Carlo or Laplace method.

C.1 Identifiability Issues

In MSE, it is quite common to encounter identifiability issues with data available from a small number of dependent sources. To deal with such issues, some restrictions on parameters are

considered in the existing models like M_{tb} and log-linear models as well as in the sample coverage approach proposed by Chao and Tsay (1998). See Section E for details. Similarly for TBM, Chatterjee and Bhuyan (2020) considered two submodels TBM-1 and TBM-2 keeping α_3 and α_4 fixed as 0, respectively. However, this process of model contraction may lead to a model representing unrealistic scenarios (Gustafson, 2005). Unlike the situation that is modeled by TBM-1, available sources related to the surveillance of humanitarian crisis are not time ordered (Chan et al., 2021), and the capture attempts are generally interdependent between themselves, including the dependence between the first and third lists (Gold et al., 2015). Similarly, TBM-2 is not suitable for the scenarios when the second-order interaction among the lists is present. The proposed THBM is intended to model more complex scenarios compared to the existing models and it also suffers from similar identifiability issues with respect to TRS data. It is important to note that a non-identifiable model may bring information about the parameters of interest, and identifiable models do not necessarily lead to better decision-making than non-identifiable ones (Gustafson, 2005; Wechsler et al., 2013). Nevertheless, it is in the best interest of the practitioners, clear guidelines should be provided that are applicable to different scenarios under consideration. In the following subsection, we propose an estimation methodology of the parameter of interest N and other associated parameters of the THBM based on the MCEM algorithm which provides a generic solution to the identifiability issue.

C.2 MCEM Algorithm

As discussed before, the likelihood function $L(\boldsymbol{\theta}|\boldsymbol{x})$, given by (C.0.2), is not mathematically tractable due to involvement of an integral and the additive structure of the cell probabilities p_{ijk} as a function of the model parameters. To simplify the likelihood function we adopt data augmentation strategy proposed by Tanner and Wong (1987). First, we partition the observed cell counts x_{ijk} depending on the various types of *list-dependence* as described in the model (1) in the main text, and define a vector of latent cell counts as

$$\boldsymbol{y} = \left\{ y_{ijk,u} : \sum_{u=1}^{\nu} y_{ijk,u} = x_{ijk}; i, j, k = 0, 1; \nu = 5^{\omega} 3^{1-\omega}; \omega = \mathbb{I}[i = j = k] \right\},$$

where $\mathbb{I}[\cdot]$ is an indicator function. For example, x_{111} can arise from all the five types of dependence structures, whereas x_{110} can be generated only based on the first and last types

of dependence structures, as presented in (1) in the main text. We also treat the capture probabilities $\boldsymbol{\mathcal{P}} = (\mathcal{P}_1, \mathcal{P}_2, \mathcal{P}_3)$ as unobserved data. Therefore, the likelihood function of $\boldsymbol{\theta}$, based on the complete data $(\boldsymbol{x}, \boldsymbol{y}, \boldsymbol{\mathcal{P}})$, is given by

$$\mathcal{L}_c(\boldsymbol{\theta}|\boldsymbol{x}, \boldsymbol{y}, \boldsymbol{\mathcal{P}}) = \{K(\boldsymbol{y})\}^{-1} \mathcal{L}_c(N, \boldsymbol{\alpha}, \boldsymbol{\mathcal{P}}|\boldsymbol{x}, \boldsymbol{y}) \times \left\{ \prod_{s=1}^{3} g_{\mathcal{P}_s}(\mathcal{P}_s|\delta_s) \right\}, \tag{C.2.1}$$

where

$$\begin{aligned}
\mathcal{L}_c(N, \boldsymbol{\alpha}, \boldsymbol{\mathcal{P}}|\boldsymbol{x}, \boldsymbol{y}) \propto\ & \frac{N!}{(N-x_0)!}[(1-\alpha_0)\mathcal{P}_1\mathcal{P}_2\mathcal{P}_3]^{y_{111,1}} \times [\alpha_1\mathcal{P}_1\mathcal{P}_3]^{y_{111,2}} \times [\alpha_2\mathcal{P}_1\mathcal{P}_2]^{y_{111,3}} \times [\alpha_3\mathcal{P}_1\mathcal{P}_2]^{y_{111,4}} \\
& \times[\alpha_4\mathcal{P}_1]^{x_{111}-\sum_{i=1}^4 y_{111,i}} \times [(1-\alpha_0)\mathcal{P}_1\mathcal{P}_2(1-\mathcal{P}_3)]^{y_{110,1}} \times [\alpha_1\mathcal{P}_1(1-\mathcal{P}_3)]^{x_{110}-y_{110,1}} \\
& \times[(1-\alpha_0)(1-\mathcal{P}_1)\mathcal{P}_2\mathcal{P}_3]^{y_{011,1}} \times [\alpha_2(1-\mathcal{P}_1)\mathcal{P}_2]^{x_{011}-y_{011,1}} \\
& \times[(1-\alpha_0)\mathcal{P}_1(1-\mathcal{P}_2)(1-\mathcal{P}_3)]^{y_{100,1}} \times [\alpha_2\mathcal{P}_1(1-\mathcal{P}_2)]^{x_{100}-y_{100,1}} \\
& \times[(1-\alpha_0)\mathcal{P}_1(1-\mathcal{P}_2)\mathcal{P}_3]^{y_{101,1}} \times [\alpha_3\mathcal{P}_1(1-\mathcal{P}_2)]^{x_{101}-y_{101,1}} \\
& \times[(1-\alpha_0)(1-\mathcal{P}_1)\mathcal{P}_2(1-\mathcal{P}_3)]^{y_{010,1}} \times [\alpha_3(1-\mathcal{P}_1)\mathcal{P}_2]^{x_{010}-y_{010,1}} \\
& \times[(1-\alpha_0)(1-\mathcal{P}_1)(1-\mathcal{P}_2)\mathcal{P}_3]^{y_{001,1}} \times [\alpha_1(1-\mathcal{P}_1)\mathcal{P}_3]^{x_{001}-y_{001,1}} \\
& \times[(1-\alpha_0)(1-\mathcal{P}_1)(1-\mathcal{P}_2)(1-\mathcal{P}_3)]^{y_{000,1}} \times [\alpha_1(1-\mathcal{P}_1)(1-\mathcal{P}_3)]^{y_{000,2}} \\
& \times[\alpha_2(1-\mathcal{P}_1)(1-\mathcal{P}_2)]^{y_{000,3}} \times [\alpha_3(1-\mathcal{P}_1)(1-\mathcal{P}_2)]^{y_{000,4}} \\
& \times[\alpha_4(1-\mathcal{P}_1)]^{N-x_0-\sum_{i=1}^4 y_{000,i}},
\end{aligned}$$

and

$$\begin{aligned}
K(\boldsymbol{y}) =\ & \prod_{u=1}^{4} y_{111,u}!(x_{111} - \sum_{u=1}^{4} y_{111,u})!y_{110,1}!(x_{110} - y_{110,1})! \\
& \times y_{011,1}!(x_{011} - y_{011,1})!y_{100,1}!(x_{100} - y_{100,1})! \\
& \times y_{101,1}!(x_{101} - y_{101,1})!y_{010,1}!(x_{010} - y_{010,1})! \\
& \times y_{001,1}!(x_{001} - y_{001,1})! \prod_{u=1}^{4} y_{000,u}!(N - x_0 - \sum_{u=1}^{4} y_{000,u})!.
\end{aligned}$$

Interestingly, the complete data likelihood, given by (C.2.1), possesses a simple form as a product of the power functions of the parameters associated with the THBM. However, this likelihood function cannot be used for estimation purposes without resolving the identifiability

issues. For this purpose, we employ a similar approach as suggested by Carle and Strub (1978) to obtain a likelihood function free of the nuisance parameters. We consider $\mathcal{P}_s$'s follow independent beta distribution with shape parameters n_s and m_s, for $s = 1, 2, 3$, and obtain their estimates as $\hat{m}_1 = x_{1\cdot\cdot}$, $\hat{n}_1 = (N - x_{1\cdot\cdot})$, $\hat{m}_2 = y_{111,1} + y_{111,3} + y_{111,4} + y_{110,1} + x_{011} + x_{010}$, $\hat{n}_2 = x_{100} + x_{101} + y_{001,1} + y_{000,1} + y_{000,3} + y_{000,4}$, $\hat{m}_3 = y_{111,1} + y_{111,2} + y_{011,1} + y_{101,1} + x_{001}$ and $\hat{n}_3 = x_{110} + y_{100,1} + y_{010,1} + y_{000,1} + y_{000,2}$, by matching moments based on the observed and latent cell counts. Then plugging the corresponding density function of $\mathcal{P}_s$ in (C.2.1), we get

$$\mathcal{L}_c(\tilde{\boldsymbol{\theta}}|\boldsymbol{x}, \boldsymbol{y}, \boldsymbol{\mathcal{P}}) = \{K(\boldsymbol{y})\}^{-1} \mathcal{L}_c(N, \boldsymbol{\alpha}, \boldsymbol{\mathcal{P}}|\boldsymbol{x}, \boldsymbol{y}) \times \left\{ \prod_{s=1}^{3} \frac{\mathcal{P}_s^{\hat{m}_s - 1} [1 - \mathcal{P}_s]^{\hat{n}_s - 1}}{B(\hat{m}_s, \hat{n}_s)} \right\}, \quad \text{(C.2.2)}$$

where $\tilde{\boldsymbol{\theta}} = (N, \boldsymbol{\alpha})$. To implement the MCEM algorithm, one must be able to sample from the conditional distributions of $\boldsymbol{y}$ given $(\boldsymbol{x}, N, \boldsymbol{\alpha}, \boldsymbol{\mathcal{P}})$, and $\boldsymbol{\mathcal{P}}$ given $(\boldsymbol{x}, \boldsymbol{y}, N, \boldsymbol{\alpha})$, denoted by $\pi(\boldsymbol{y}|\boldsymbol{x}, N, \boldsymbol{\alpha}, \boldsymbol{\mathcal{P}})$, and $\pi(\boldsymbol{\mathcal{P}}|\boldsymbol{x}, \boldsymbol{y}, N, \boldsymbol{\alpha})$, respectively. Note that $\pi(\boldsymbol{\mathcal{P}}|\boldsymbol{x}, \boldsymbol{y}, N, \boldsymbol{\alpha})$ is expressed as the product of Beta distributions, and $\pi(\boldsymbol{y}|\boldsymbol{x}, N, \boldsymbol{\alpha}, \boldsymbol{\mathcal{P}})$ is expressed as a product of Multinomial and Binomial distributions (see Section D for details). This approach is close in spirit to the method of *EM-within-Gibbs* and stochastic *EM-within-Gibbs* considered by Chatterjee and Mukherjee (2016a). Now, we employ a simple iterative algorithm to estimate the model parameters using the following steps.

Step 1. Set $t = 0$ and initialize $(\boldsymbol{y}^{(t)}, N^{(t)}, \boldsymbol{\alpha}^{(t)})$.

Step 2. Generate K samples $\boldsymbol{\mathcal{P}_i}^{(t+1)}$ from $\pi(\boldsymbol{\mathcal{P}}|\boldsymbol{x}, \boldsymbol{y}^{(t)}, N^{(t)}, \boldsymbol{\alpha}^{(t)})$, and then $\boldsymbol{y}_i^{(t+1)}$ from $\pi(\boldsymbol{y}|\boldsymbol{x}, N^{(t)}, \boldsymbol{\alpha}^{(t)}, \boldsymbol{\mathcal{P}}^{(t+1)})$ for $i = 1, \ldots, K$.

Step 3. E step: Monte Carlo approximation of (C.2.2) is computed as

$$\frac{1}{K} \sum_{i=1}^{K} \log \mathcal{L}_c(\tilde{\boldsymbol{\theta}}|\boldsymbol{x}, \boldsymbol{y}_i^{(t+1)}, \boldsymbol{\mathcal{P}}_i^{(t+1)})$$

Step 4. M Step: $\underset{\tilde{\boldsymbol{\theta}}^{(t+1)}}{\operatorname{argmax}} \quad \frac{1}{K} \sum_{i=1}^{K} \log \mathcal{L}_c(\tilde{\boldsymbol{\theta}}|\boldsymbol{x}, \boldsymbol{y}_i^{(t+1)}, \boldsymbol{\mathcal{P}}_i^{(t+1)})$

Step 5. Repeat Step 2-4 until convergence of $\left\{\tilde{\boldsymbol{\theta}}^{(t)}\right\}_{t \geq 0}$, i.e. until $\|\tilde{\boldsymbol{\theta}}^{(t)} - \tilde{\boldsymbol{\theta}}^{(t-1)}\| < \epsilon$, for some prespecified threshold $\epsilon > 0$.

The problem of multimodality of the likelihood often arises when fitting models with latent variables (Bartolucci and Pennoni, 2007). We generate different initial values randomly for the first step in the aforementioned algorithm. We take the parameter vector that at convergence gives the highest value of $\mathcal{L}_c(\tilde{\boldsymbol{\theta}}|\boldsymbol{x}, \boldsymbol{y}, \boldsymbol{\mathcal{P}})$, given in C.2.2, as an estimate of θ, denoted by $\tilde{\theta}$.

D Derivations of Distributions of the Latent Cell Counts

The conditional density of the latent cell counts $\boldsymbol{y}$, given $(N, \boldsymbol{\alpha}, \boldsymbol{\mathcal{P}}, \boldsymbol{x})$, is easily obtained from (4) as

$$\pi(\boldsymbol{y}|N, \boldsymbol{\alpha}, \boldsymbol{\mathcal{P}}, \boldsymbol{x}) \propto \prod_{i,j,k=0,1} \pi(y_{ijk}|\boldsymbol{\theta}, \boldsymbol{\mathcal{P}}, \boldsymbol{x}),$$

where

$$\begin{aligned}
\pi(\boldsymbol{y_{111}}|\boldsymbol{\theta}, \boldsymbol{x}) &\propto Multinomial(x_{111}; Q^*_{111,1}, Q^*_{111,2}, Q^*_{111,3}, Q^*_{111,4}, Q^*_{111,5}),\\
\pi(y_{110,1}|\boldsymbol{\theta}, \boldsymbol{x}) &\propto Binomial(x_{110}; (1-\alpha_0)\mathcal{P}_1\mathcal{P}_2(1-\mathcal{P}_3)),\\
\pi(y_{011,1}|\boldsymbol{\theta}, \boldsymbol{x}) &\propto Binomial(x_{011}; (1-\alpha_0)(1-\mathcal{P}_1)\mathcal{P}_2\mathcal{P}_3),\\
\pi(y_{100,1}|\boldsymbol{\theta}, \boldsymbol{x}) &\propto Binomial(x_{100}; (1-\alpha_0)\mathcal{P}_1(1-\mathcal{P}_2)(1-\mathcal{P}_3)),\\
\pi(y_{101,1}|\boldsymbol{\theta}, \boldsymbol{x}) &\propto Binomial(x_{101}; (1-\alpha_0)\mathcal{P}_1(1-\mathcal{P}_2)\mathcal{P}_3),\\
\pi(y_{010,1}|\boldsymbol{\theta}, \boldsymbol{x}) &\propto Binomial(x_{010}; (1-\alpha_0)(1-\mathcal{P}_1)\mathcal{P}_2(1-\mathcal{P}_3)),\\
\pi(y_{001,1}|\boldsymbol{\theta}, \boldsymbol{x}) &\propto Binomial\left(x_{001}; (1-\alpha_0)(1-\mathcal{P}_1)(1-\mathcal{P}_2)\mathcal{P}_3\right),\\
\pi(\boldsymbol{y_{000}}|\boldsymbol{\theta}, \boldsymbol{x}) &\propto Multinomial(N - x_0; Q^*_{000,1}, Q^*_{000,2}, Q^*_{000,3}, Q^*_{000,4}, Q^*_{000,5}),
\end{aligned}$$

with $\boldsymbol{y_{111}} = (y_{111,1}, y_{111,2}, y_{111,3}, y_{111,4}, y_{111,5})$, $\boldsymbol{y_{000}} = (y_{000,1}, y_{000,2}, y_{000,3}, y_{000,4}, y_{000,5})$, $Q^*_{111,1} = (1-\alpha_0)\mathcal{P}_1\mathcal{P}_2\mathcal{P}_3/Q_{111}$, $Q^*_{111,2} = \alpha_1\mathcal{P}_1\mathcal{P}_3/Q_{111}$, $Q^*_{111,3} = \alpha_2\mathcal{P}_1\mathcal{P}_2/Q_{111}$, $Q^*_{111,4} = \alpha_3\mathcal{P}_1\mathcal{P}_2/Q_{111}$, $Q^*_{111,5} = \alpha_4\mathcal{P}_1/Q_{111}$, $Q_{111} = (1-\alpha_0)\mathcal{P}_1\mathcal{P}_2\mathcal{P}_3 + \alpha_1\mathcal{P}_1\mathcal{P}_3 + \alpha_2\mathcal{P}_1\mathcal{P}_2 + \alpha_3\mathcal{P}_1\mathcal{P}_2 + \alpha_4\mathcal{P}_1$; $Q^*_{000,1} = (1-\alpha_0)(1-\mathcal{P}_1)(1-\mathcal{P}_2)(1-\mathcal{P}_3)/Q_{000}$, $Q^*_{000,2} = \alpha_1(1-\mathcal{P}_1)(1-\mathcal{P}_3)/Q_{000}$, $Q^*_{000,3} = \alpha_2(1-\mathcal{P}_1)(1-\mathcal{P}_2)/Q_{000}$, $Q^*_{000,4} = \alpha_3(1-\mathcal{P}_1)(1-\mathcal{P}_2)/Q_{000}$, $Q^*_{000,5} = \alpha_4(1-\mathcal{P}_1)/Q_{000}$, $Q_{000} = (1-\alpha_0)(1-\mathcal{P}_1)(1-\mathcal{P}_2)(1-\mathcal{P}_3) + \alpha_1(1-\mathcal{P}_1)(1-\mathcal{P}_3) + \alpha_2(1-\mathcal{P}_1)(1-\mathcal{P}_2) + \alpha_3(1-\mathcal{P}_1)(1-\mathcal{P}_2) + \alpha_4(1-\mathcal{P}_1)$.

E Existing Models and Estimates

In this section, we present frequently used models for TRS and associated estimates for the population size. These models incorporate *individual heterogeneity* and/or *list-dependence*. The assumptions associated with these models and their limitations in real applications are also discussed briefly. We consider these estimates to compare the performance of the proposed method in the section on Simulation Study in the main article.

E.1 Log-linear Model

Fienberg (1972b) and Bishop et al. (1975) discussed several log-linear models (LLMs) to account for *list-dependence* using interaction effects. The general LLM under TRS is given by

$$\log(m_{ijk}) = u_0 + u_{1(i)} + u_{2(j)} + u_{3(k)} + u_{12(ij)} + u_{13(ik)} + u_{23(ik)} + u_{123(ijk)}, \quad \text{(E.1.1)}$$

where $m_{ijk} = \mathbb{E}[x_{ijk}]$, $u_{l(0)} + u_{l(1)} = 0$, $u_{ll'(0j)} + u_{ll'(1j)} = 0$, $u_{ll'(i0)} + u_{ll'(i1)} = 0$, $u_{ll'l^*(0jk)} + u_{ll'l^*(1jk)} = 0$, $u_{ll'l^*(i0k)} + u_{ll'l^*(i1k)} = 0$, $u_{ll'l^*(ij0)} + u_{ll'l^*(ij1)} = 0$, for all $l, l', l^* = 1, 2, 3$, with $l \neq l' \neq l^*$ (Bishop et al., 1975, p.64). The parameters u_l, $u_{ll'}$ and u_{123} denote the main effects, pairwise interaction effects, and the second order interaction effect for $l, l' = 1, 2, 3$. See Fienberg (1972a) and Fienberg (1972b) for more details. To ensure the estimability of the model given in (E.1.1), one needs to consider $u_{123} = 0$, i.e. no second-order interaction between lists. Under this assumption, the estimate of m_{000} is given by

$$\hat{m}_{000} = \frac{\hat{m}_{111}\hat{m}_{001}\hat{m}_{100}\hat{m}_{010}}{\hat{m}_{101}\hat{m}_{011}\hat{m}_{110}},$$

where $\hat{m}_{ijk}$ is the maximum likelihood estimate of m_{ijk} assuming x_{ijk} to be a realization of an independent Poisson random variate for all (i, j, k)th cells, except the $(0, 0, 0)$th cell. Finally, the estimate of N is obtained as $\hat{N}_{LLM} = n + \hat{m}_{000}$ (Fienberg, 1972b). This is equivalent to the estimator proposed by Zaslavsky and Wolfgang (1993) with $\alpha_{EPA} = 1$. With the additional assumption that $u_{12} = u_{13} = u_{23} = 0$, LLM in (E.1.1) reduces to the Independent Model (IM) which has no closed-form solution (Fienberg, 1972b).

E.2 Time Behavioural Response Variation Model: M_{tb}

It is often observed in TRS that an individual's behavior changes in subsequent recapture attempts after the initial attempt. This change is known as behavioral response variation.

When this behavioral response variation is considered along with the assumption of list variation in capture probabilities, one would have a model known as M_{tb} model (Otis et al., 1978; Chatterjee and Mukherjee, 2016b). However, M_{tb} model does not account for the *individual heterogeneity* in capture probabilities.

Denote the first-time capture probability of any individual in the l-th list is by f_s for $s = 1, 2, 3$, and the recapture probability is denoted by c_s for $s = 2, 3$. Further, u_s and m_s denote, respectively, the number of first-time captured and recaptured individuals in the s-th list. Therefore, based on the the sufficient statistics ($u_1 = x_{1\cdot\cdot}, u_2 = x_{01\cdot}, u_3 = x_{001}, m_2 = x_{11\cdot}, m_3 = x_{101} + x_{011} + x_{111}$) and the assumption of constant proportionality, i.e., $c_s/f_s = \phi$, for $s = 2, 3$, likelihood of the model M_{tb} is given by

$$L(N, \boldsymbol{f}, \phi) = \frac{N!}{(N-n)} f_1^{u_1}(1-f_1)^{N-u_1}\phi^{m_2+m_3}\prod_{s=2}^{3} f_s^{u_s+m_s}(1-f_s)^{N-M_{s+1}}(1-\phi p_s)^{M_s-m_s},$$

where $n = \sum_{i,j,k:ijk\neq 000} x_{ijk}$ and $M_s = u_1 + u_2 + ... + u_{s-1}$ denote the total number of distinct captured individuals and the number of individuals captured at least once prior to the s-th attempt, respectively (Chatterjee and Bhuyan, 2020). In this context, Chao et al. (2000) discussed various likelihood-based methods for the estimation of N. Note that, the M_{tb} model does not utilize full information available from seven known cells in TRS, and hence, it loses efficiency in order to estimate N (Chatterjee and Bhuyan, 2020). Moreover, the assumption of $c_2/f_2 = c_3/f_3 = \phi$ is not justifiable in various real-life applications.

E.3 Models with individual heterogeneity

In educational statistics and Psychometry, Rasch model (Rasch, 1961) is widely is used to account for the *individual heterogeneity* and list variation. The capture probabilities are model using logistic regression:

$$\log\left[\frac{\mathbb{E}[Z_h^{(l)}]}{1-\mathbb{E}[Z_h^{(l)}]}\right] = v_h + w_s, \qquad h = 1, 2, \cdots, N; \quad s = 1, 2, 3, \tag{E.3.1}$$

where v_h is a random effect representing the catchability of hth individual and w_s is list-variation effect of sth list. Even with the absence of behavioural response variation, dependence

among the lists are induced is purely due to *individual heterogeneity* (Chao et al., 2001). When we set $v_h = 0$ in (E.3.1), the model reduces to a multiple-recapture model with list independence. We now represent the Rasch model as a mixed-effects generalized linear model that allows for *individual heterogeneity* and list variation in the following subsections.

E.3.1 Quasi-symmetry Model

The marginal cell probabilities for all the eight cells in a TRS can be represented as:

$$\log(p_{ijk}) = a + iw_1 + jw_2 + kw_3 + \gamma(i_+), \tag{E.3.2}$$

where $i_+ = i + j + k$ and $\gamma(t) = \log\left[\mathbb{E}\left[\exp(v_h t)|i = j = k = 0\right]\right]$. See Darroch et al. (1993); Fienberg et al. (1999) for detailed derivation. Note that the term $\gamma(i_+)$ is invariant with respect to permutations of (i, j, k), and hence the model in (E.3.2) is known as quasi-symmetry model (QSM) (Darroch, 1981). This is equivalent to the following assumption involving two constraints

$$p_{011}p_{100} = p_{101}p_{010} = p_{110}p_{001} \tag{E.3.3}$$

which do not involve p_{000}. Therefore, an additional assumption is required, such as no second-order interaction exists. Under this assumption, the estimated count of the $(0, 0, 0)$th cell is given by

$$\hat{x}_{000} = \frac{\hat{x}_{111}\hat{x}_{100}\hat{x}_{010}\hat{x}_{001}}{\hat{x}_{110}\hat{x}_{101}\hat{x}_{011}},$$

where $\hat{x}_{ijk}$ is the maximum likelihood estimate of $\mathbb{E}[x_{ijk}|n]$ (Darroch et al., 1993).

E.3.2 Partial Quasi-symmetry Model

In many situations, the constraint provided in (E.3.3) associated with quasi-symmetry model is not realistic. However, it is reasonable to assume $p_{011}p_{100} = p_{101}p_{010}$ if $x_{011}x_{100}$ and $x_{101}x_{010}$ are close enough (Darroch et al., 1993). This condition implies that the patterns of *individual heterogeneity* are different for two different group of sources or lists as considered in generalized Rasch model proposed by Stegelman (1983). Under this assumption, the marginal cell probabilities can be represented as:

$$\log(p_{ijk}) = a + iw_1 + jw_2 + kw_3 + \gamma(i + j, k),$$

where $\gamma(t_1, t_2) = \log\left[\mathbb{E}\left[\exp(v_h t_1)\exp(z_h t_2)|i = j = k = 0\right]\right]$ with two nonidentiacally distributed random effects v_h and z_h associated with group of lists (L_1, L_2) and L_3, respectively . This model in is known as partial quasi-symmetry model (PQSM). See Darroch et al. (1993); Fienberg et al. (1999) for more details.

E.4 Sample Coverage Method

The idea of the sample coverage of a given sample is the probability weighted fraction of the population captured in that sample. The main principle relies on the fact that it is difficult to estimate the number of undercount directly, but the sample coverage can be well estimated in any non-independent situation with heterogeneous capture probability (Chao and Tsay, 1998). In the context of TRS, the sample coverage is defined based on three hypothetical populations I, II, and III with capture probabilities $\left\{\mathbb{E}\left[Z_h^{(1)} \mid Z_h^{(2)}, Z_h^{(3)}\right]; h = 1, \ldots, N\right\}$, $\left\{\mathbb{E}\left[Z_h^{(2)} \mid Z_h^{(1)}, Z_h^{(3)}\right]; h = 1, \ldots, N\right\}$, and $\left\{\mathbb{E}\left[Z_h^{(3)} \mid Z_h^{(1)}, Z_h^{(2)}\right]; h = 1, \ldots, N\right\}$ corresponding to lists L_1, L_2, and L_3, respectively. In particular, the sample coverage of first two lists $L_1 \cup L_2$ with respect to the population III is given by

$$C_{III}(L_1 \cup L_2) = \frac{\sum_{h=1}^{N} \mathbb{E}\left[Z_h^{(3)} \mid Z_h^{(1)}, Z_h^{(2)}\right] \mathbb{I}[Z_h^{(1)} + Z_h^{(2)} > 0]}{\sum_{h=1}^{N} \mathbb{E}\left[Z_h^{(3)} \mid Z_h^{(1)}, Z_h^{(2)}\right]}.$$

Similarly, one can obtain the sample coverages of other two possibles combinations of samples, $C_{II}(L_1 \cup L_3)$ and $C_I(L_2 \cup L_3)$ with respect to the populations II and I, respectively. Now, the sample coverage of the three lists is defined as $C = \frac{1}{3}\left\{C_{III}(L_1 \cup L_2) + C_{II}(L_1 \cup L_3) + C_I(L_2 \cup L_3)\right\}$, and estimated by $\hat{C} = 1 - \frac{1}{3}\left[\frac{x_{100}}{n_1} + \frac{x_{010}}{n_2} + \frac{x_{001}}{n_3}\right]$. Finally, the estimator of N on based sample coverage approach is given by

$$\hat{N}_{sc} = \frac{x_{\cdot 11} + x_{1 \cdot 1} + x_{11 \cdot}}{3\hat{C}} \left\{1 - \frac{1}{3\hat{C}}\left[\frac{(x_{1 \cdot 0} + x_{\cdot 10})x_{11 \cdot}}{n_1 n_2} + \frac{(x_{10 \cdot} + x_{\cdot 01})x_{1 \cdot 1}}{n_1 n_3} + \frac{(x_{0 \cdot 1} + x_{01 \cdot})x_{\cdot 11}}{n_2 n_3}\right]\right\}^{-1}.$$

We refer this estimate as SC. From empirical studies, it is found that the performance of $\hat{N}_{sc}$ is satisfactory if the sample coverage is over 55%. The performance also depends on the remainder term involved in the derivation of $\hat{N}_{sc}$, and the bias increases with its magnitude (Chao and

Tsay, 1998). Moreover, this method may produce infeasible estimates, i.e. $\hat{N}_{sc} < x_0$, unlike other methods.

F Simulation Study

In this section, we vividly describe the simulation study and its findings presented in the *Simulation Study* section of the main text. We consider eight different compositions of populations representing varying degrees of *list-dependence* and *individual heterogeneity* in capture probabilities and denote them as P1-P8 in Table F.1. The choices of $\boldsymbol{\alpha}$ represent different degrees of *list-dependence*. The heterogeneity in capture probabilities are modeled as $b_s = \log\left[\frac{\mathcal{P}_s}{1-\mathcal{P}_s}\right]$, where b_s's are normal and generalized logistic type-I variates for $s = 1, 2, 3$. To compare the performance of the proposed estimator (THBM) with the existing competitors, we consider the following models: log-linear model (LLM) (Fienberg, 1972b), M_{tb} model (Chao et al., 2000; Chatterjee and Bhuyan, 2020), quasi-symmetry model (QSM) (Darroch et al., 1993), partial quasi-symmetry model (PQSM) (Darroch et al., 1993), non-parametric sample coverage method (SC) (Chao and Tsay, 1998) and the independent model (IM) i.e. LLM without interaction effects (Fienberg, 1972b). The details of these existing models and associated estimates are briefly presented in Section E.

Table F.1: Composition of the simulated populations.

Populations	$(\alpha_1, \alpha_2, \alpha_3, \alpha_4)$	b_1	b_2	b_3
P1	$(0.30, 0.30, 0.15, 0.10)$	$Normal(1, 5)$	$Normal(0.5, 5)$	$Normal(0, 5)$
P2	$(0.30, 0.30, 0.15, 0.10)$	$Normal(0.5, 1)$	$Normal(0.4, 1)$	$Normal(0.3, 1)$
P3	$(0.25, 0.15, 0.35, 0.10)$	$Normal(1, 5)$	$Normal(0.5, 5)$	$Normal(0, 5)$
P4	$(0.25, 0.15, 0.35, 0.10)$	$Normal(0.5, 1)$	$Normal(0.4, 1)$	$Normal(0.3, 1)$
P5	$(0.30, 0.30, 0.15, 0.10)$	$GL_I(1)$	$GL_I(1.4)$	$GL_I(1.8)$
P6	$(0.30, 0.30, 0.15, 0.10)$	$GL_I(1.6)$	$GL_I(1.2)$	$GL_I(0.8)$
P7	$(0.25, 0.15, 0.35, 0.10)$	$GL_I(1)$	$GL_I(1.4)$	$GL_I(1.8)$
P8	$(0.25, 0.15, 0.35, 0.10)$	$GL_I(1.6)$	$GL_I(1.2)$	$GL_I(0.8)$

$Normal(\mu, \sigma)$ denotes normal distribution with mean μ and standard deviation σ.
$GL_I(\eta)$ denotes generalized logistic type-I distribution with parameter η.

We generate 1000 datasets from the proposed model keeping the total population size N

fixed at 500 and 1000 for each of these eight different choices of populations P1-P8 (see Table F.1), and compute the relative mean absolute error as $RMAE = \frac{1}{1000}\sum_{r=1}^{1000}|\frac{\hat{N}_r - N}{N}|$, where $\hat{N}_r$ denotes the estimate based on dataset generated at the rth replication, for $r = 1, \ldots, 1000$. The comparison of different estimators with respect to RMAE is presented in Figure F.1. In the capture-recapture setting, point estimators of population size commonly possess positively skewed distributions and we also observed a similar pattern in our study. Therefore, we obtain 95% interval (C.I.) for N based on the log-transformation method, proposed by Chao (1987). In this method, $\log(\hat{N} - n)$ is approximately treated as normal variate and that gives 95% confidence interval for N as $[n + (\hat{N} - n)/C, n + (\hat{N} - n)C]$, where $C = \exp\left[1.96 \times \left[\log(1 + \hat{\sigma}^2_{\hat{N}}/(\hat{N} - n)^2)\right]^{1/2}\right]$, and $\hat{\sigma}^2_{\hat{N}}$ is the estimate of the variance of the estimator $\hat{N}$, computed based on 1000 nonparametric bootstrap samples which are drawn from implicitly assumed multinomial distribution for the mark-recapture model (Buckland and Garthwaite, 1991). Following Chatterjee and Mukherjee (2018), we compute the length of the 95% confidence interval (LCI) as well as its coverage probability (CP) and represent both the results in Figure F.2, where height and width of each bar represent CP and LCI of the corresponding method, respectively.

The proposed model performs the best in terms of RMAE for all the populations P1-P8 for both $N = 500$ and 1000. Moreover, the CPs of the proposed THBM estimator are higher than all other competitors. The CPs of M_{tb} are similar to THBM but CIs are very wide due to high variability. However, the LCIs of the proposed THBM estimate are the smallest in all the cases under consideration except P5 and P7. As expected, the RMAEs of the IM are the highest among all the estimators for most of the populations. The performance of LLM, QSM and PQSM estimators are comparable with respect to both RMAE and CP. But the M_{tb} performs better than LLM, QSM and PQSM. It is important to note that the CPs of SC and IM are very low as the associated CIs are very tight. The performances of each estimator are similar over all the populations with respect to CPs and LCIs except P5 and P7, where the LCIs are comparably wider. We also observe that the SC estimate suffers from boundary problems (see Section E.4 for details) in our simulation study. One can easily configure populations to generate data such that the percentage of infeasible estimates obtained from SC method can be as high as 60% to 80%. Also, the QSM, PQSM, and LLM may fail to converge in some situations and produce extremely large estimates. We have not considered those cases here for

a valid and fair comparison. In some scenarios, sample size (i.e. the sum of all observed cell counts) may be small due to low capture probabilities across the sources. Unlike some previous work (Nour, 1982), our proposed method does not require any restriction on the domains of capture probabilities of individuals. Hence, the proposed method can definitely be applied when the sample size is small. To investigate, we conducted simulation studies where the sample size is less than 50% of the true population size. For example, we generate the random effects b_i's from generalized logistic type-I distribution with parameter 0.5 with dependence parameters $(\alpha_1, \alpha_2, \alpha_3, \alpha_4) = (0.2, 0.15, 0.4, 0.1)$ and the resulting RMAE is approximately 0.055. As expected, the RMAE increases compared to the cases where the sample size is not too small. However, the relative performance of the proposed method compared to the existing competitors remains superior.

F.1 Sensitivity of Model Misspecification

To study the effect of model misspecification when the data-generating mechanism deviates from the fitted model, we analyse the sensitivity of the estimates based on the proposed THBM in comparison with the existing estimators. For this purpose, we generate the latent capture statuses of the individuals X_{1h}, X_{2h}, X_{3h} from Bernoulli distributions with respective probabilities $P_h^{(1)}$, $P_h^{(2)}$, and $P_h^{(3)}$, where $P_h^{(j)} = \min\{P_h^{(j-1)}\mathbb{I}[Z_h^{(j-1)} = 0] + 1.2\mathbb{I}[Z_h^{(j-1)} = 1], 0.99\}$, for $j = 2, 3$, and $h = 1, \ldots, N$, and then generate $Z_h^{(1)}$, $Z_h^{(2)}$, $Z_h^{(3)}$ using the model in Equation (1) in the main text with $\boldsymbol{\alpha} = (0.1, 0.1, 0.1, 0.1)$. This mechanism induces an additional dependence among the capture statuses similar to a first-order auto-regressive model as considered in Chao (1987). To incorporate heterogeneity in the capture probabilities, we generate $P_h^{(l)}$ from two different choices of probability distributions S1: $Beta(2, 2)$, and S2: $Beta(2, 4)$ for $s = 1, 2, 3$. We also generate data from Rasch model (Rasch, 1961) given by Equation (E.3.1) in Section E.3, where the capture status of the h-th individual in List l, $Z_h^{(s)}$ is a Bernoulli variate with probability $P_h^{(s)} = \frac{\exp[v_h + w_s]}{1+\exp[v_h + w_s]}$, for $h = 1, \ldots, N$, $s = 1, 2, 3$, with v_h's as standard normal variates and two different choices of the fixed effects S3: $(s_1, s_2, s_3) = (-1, 0, 1)$, and S4: $(s_1, s_2, s_3) = (1, 0.5, 0.1)$. Note that all the aforementioned configurations (S1, S2, S3, S4) for data generation completely deviates from the assumed structure of the fitted models. The resulting RMAEs from the proposed THBM and its competitors are plotted in Figure F.3. Following a similar mechanism as discussed in Section F, we present CP and LCI of the

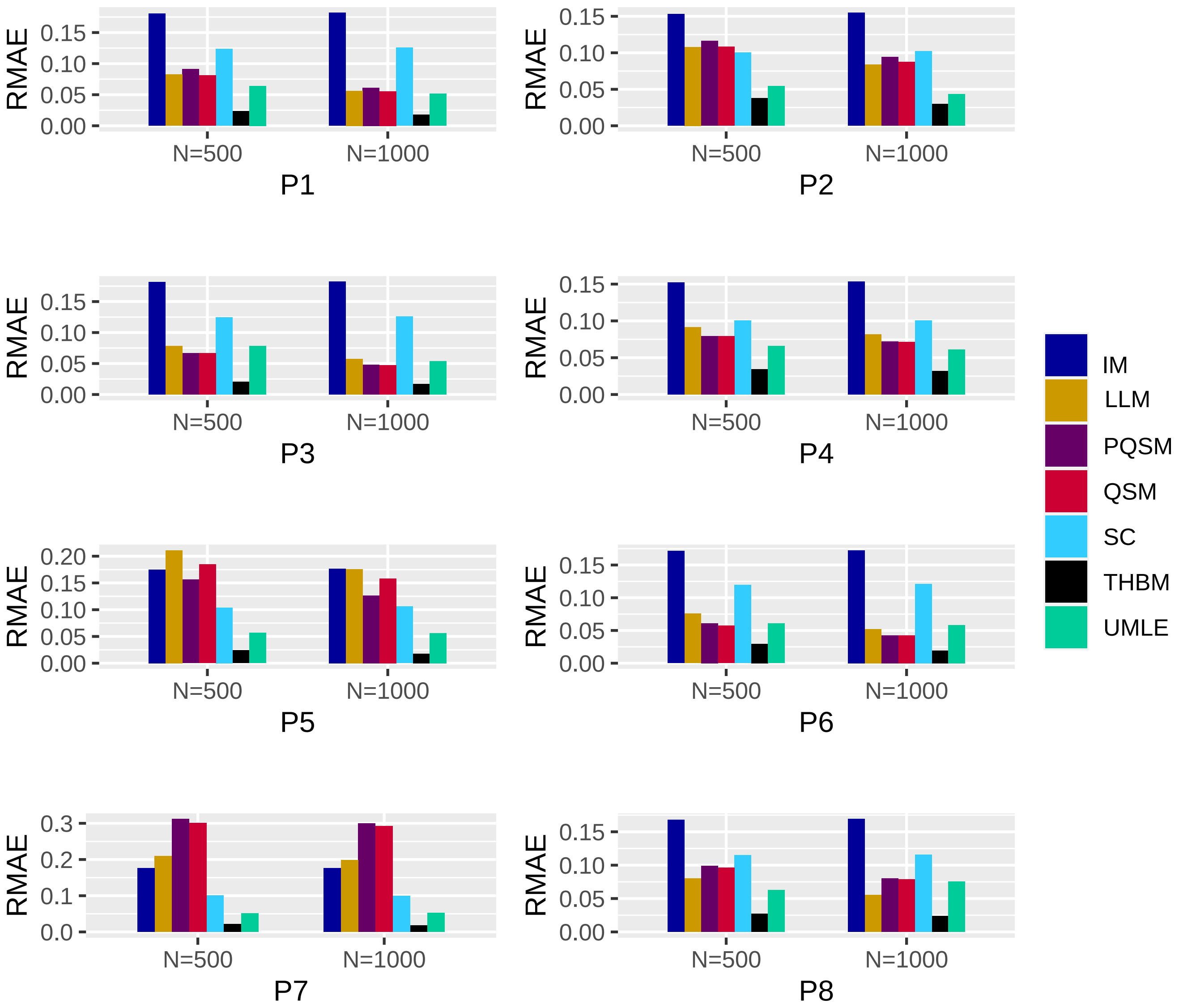

Figure F.1: Comparison of RMAEs of the estimators for populations P1-P8.

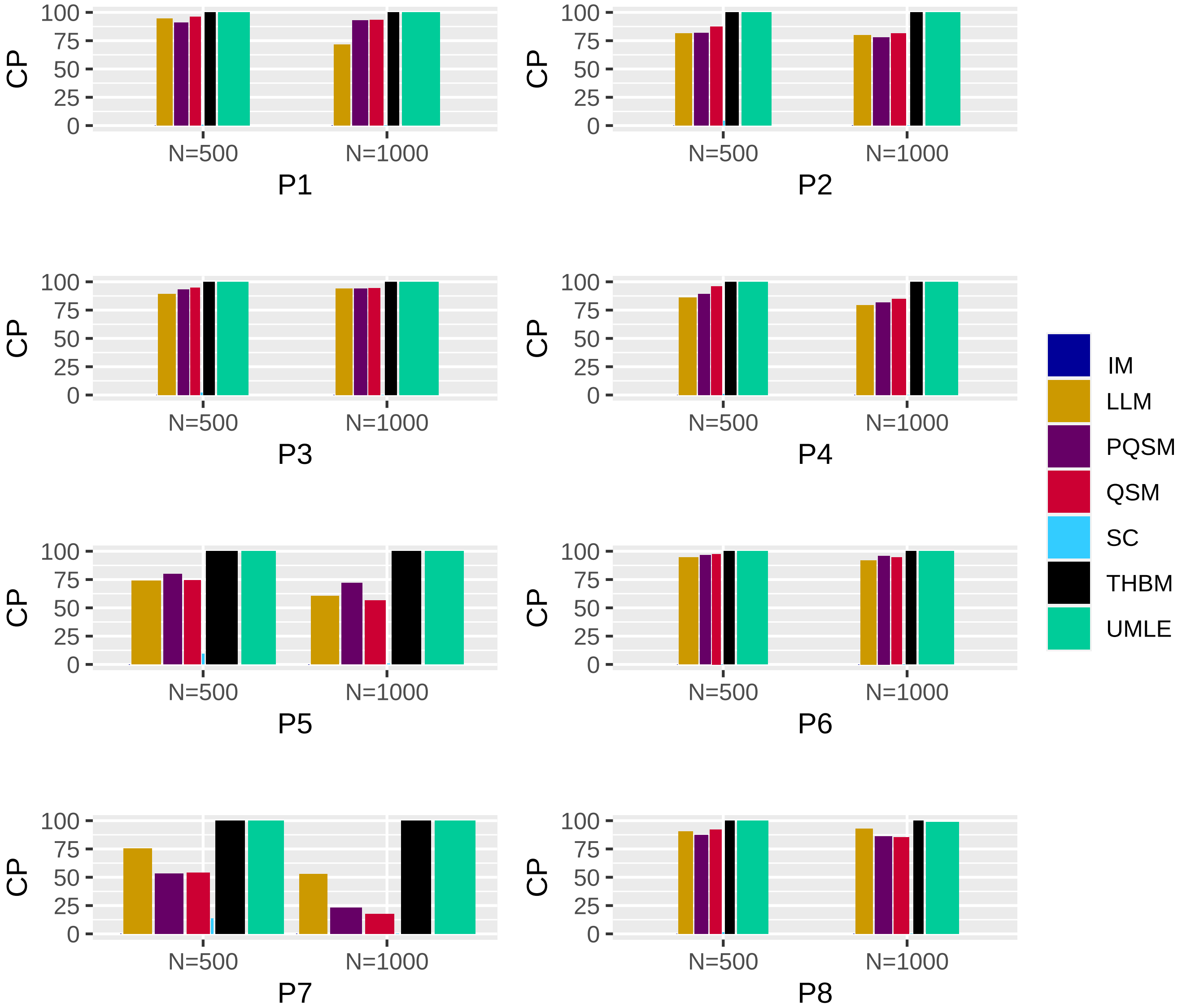

Figure F.2: Comparison of CPs (in %) and the relative LCIs of the estimators, represented by the bar height and bar width, respectively, for populations P1-P8.

proposed and its competitors in Figure F.4. Here also, the proposed estimator outperforms the existing competitors with respect to RMAE as discussed in Section F. Overall, the CPs of the proposed estimator and M_{tb} model are close to 100% and much better than all other estimators. In particular, the performance of SC is very poor with respect to RMAEs and CPs. As expected, the performance of the model IM is the worst in all respects.

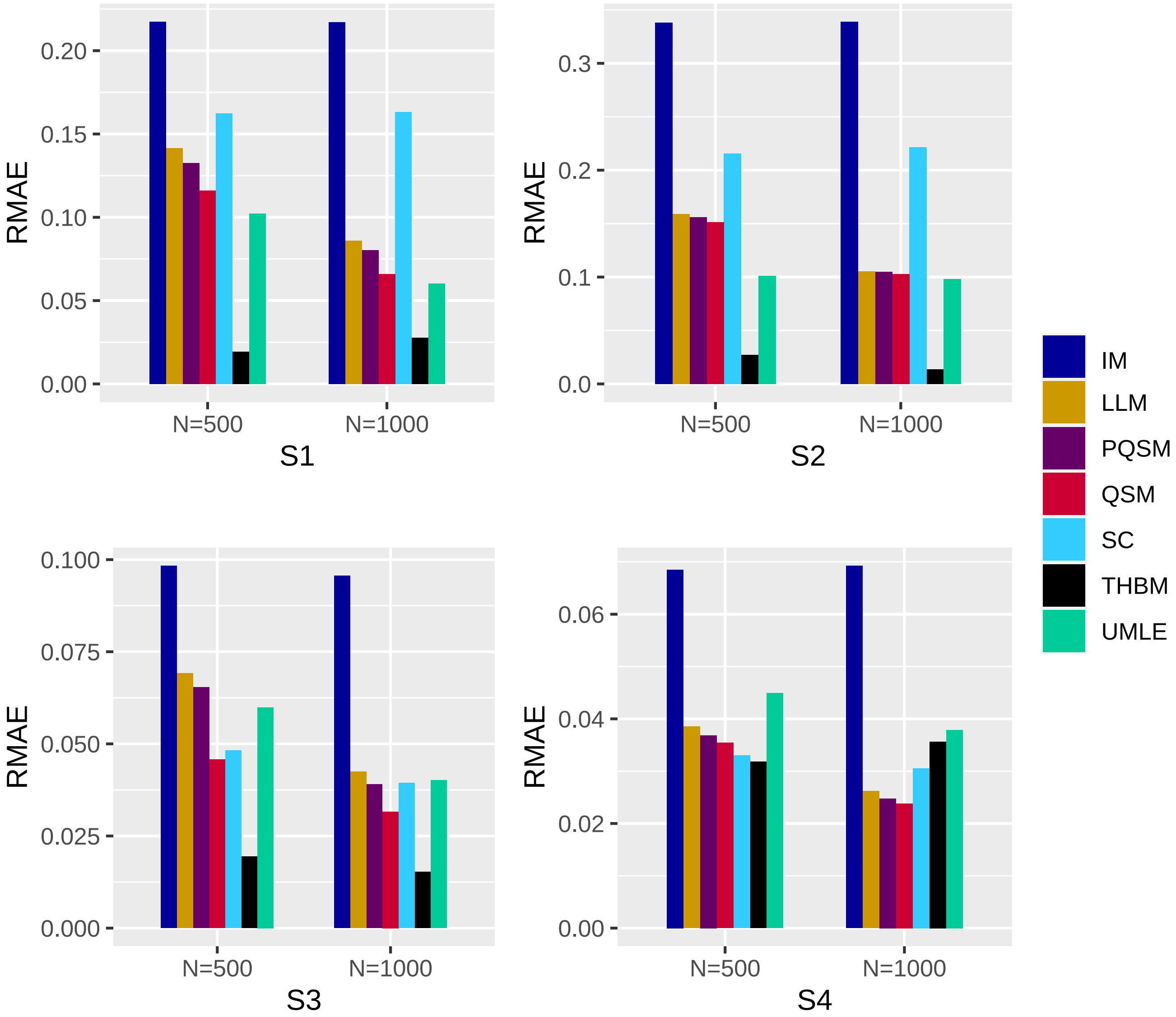

Figure F.3: Comparison of the RMAEs of the proposed estimator with the existing competitors under model misspecification when data generated from configurations S1-S4.

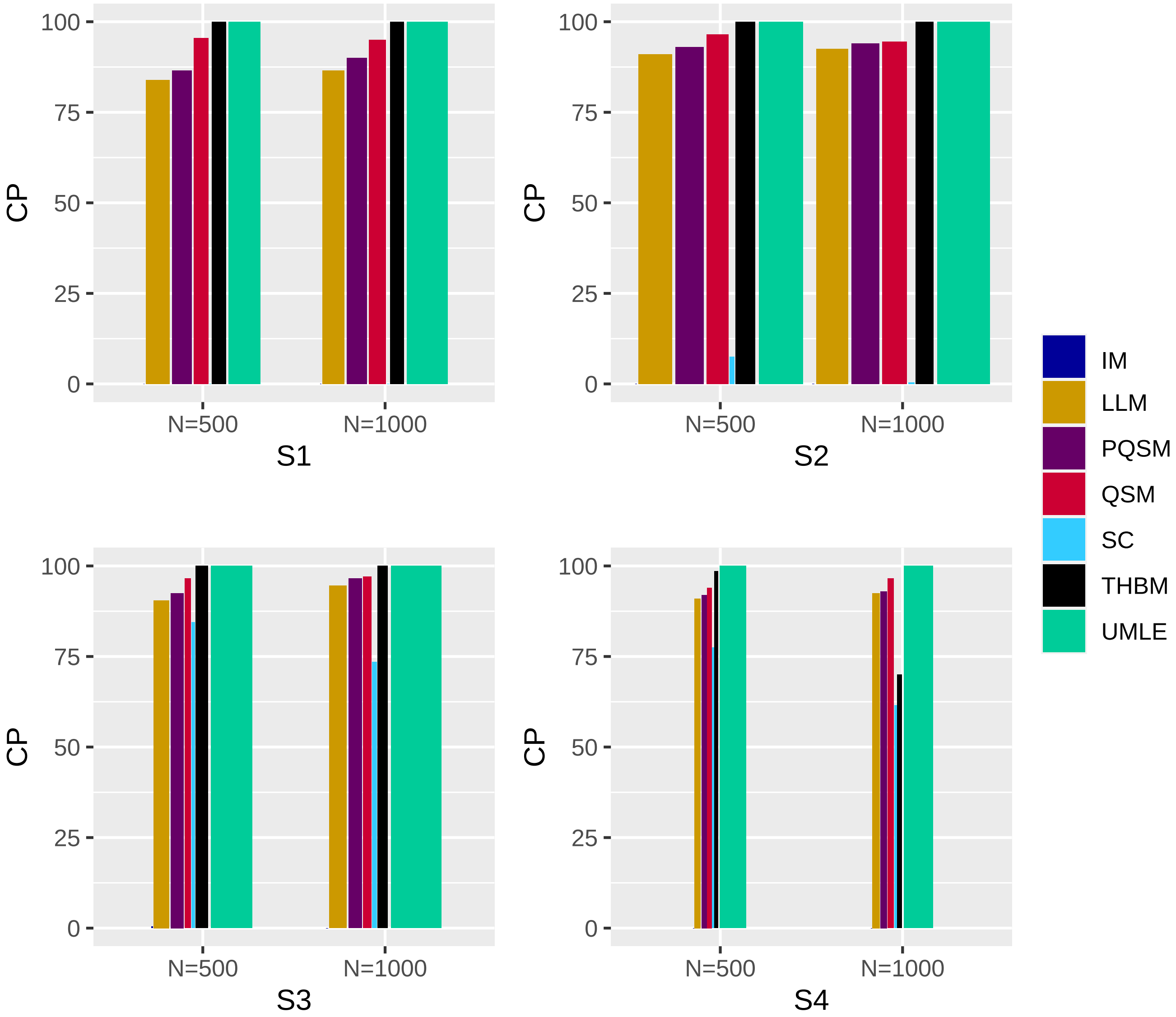

Figure F.4: Comparison of the CPs (in %) of the proposed estimator with the existing competitors under model misspecification when data generated from configurations S1-S4.

G Data Analysis

Table G.2: Summary results of data analysis on the ALS disease surveillance during 1991 Gulf War and WTC Terrorists attacks in 2001.

	THBM	SC	QSM	PQSM	LLM	M_{tb}
			Gulf War Deployed Veterans			
$\hat{N}$	53	44	43	45	45	40
CI	(42, 86)	(36, 52)	(38, 79)	-	(36, 70)	(40, 88)
			Gulf War Non-deployed Veterans			
$\hat{N}$	78	74	70	72	72	134
CI	(68, 107)	(69, 98)	(67, 93)	(66, 162)	-	(68, 478)
			WTC Terrorists attack			
$\hat{N}$	13919	11977	11906	14698	12124	8974
CI	(13733, 14112)	(11203, 12718)	(11266, 12571)	(13015, 16092)	(10812, 12397)	(8973, 9197)

THBM: Trivariate Heterogeneous Bernoulli Model; SC: Sample Coverage; QSM: Quasi-symmetry Model; PQSM: Partial Quasi-symmetry Model; LLM: Log-linear Model, M_{tb}: Time Behavioural Response Variation Model

H R Programme

The R programme for data analysis based on the proposed methodology is available in the following link. https://github.com/Prajamitra/Estimating-Prevalence-MCEM/blob/main/MCEM_Code.R

References

Baillargeon, S. and Rivest, L.-P. (2007). Rcapture: Loglinear models for capture-recapture in r. *Journal of Statistical Software*, 19:1–31. 2

Bartolucci, F. and Pennoni, F. (2007). A class of latent markov models for capture–recapture data allowing for time, heterogeneity, and behavior effect. *Biometrics*, 63:568–578. 8

Bishop, Y. M. M., Fienberg, S. E., and Holland, P. W. (1975). *Discrete multivariate analysis: Theory and practice*. Cambridge, MA: The MIT Press. 9

Buckland, S. T. and Garthwaite, P. H. (1991). Quantifying precision of mark-recapture estimates using the bootstrap and related methods. *Biometrics*, 47:255–268. 14

Carle, F. L. and Strub, M. R. (1978). A new method for estimating population size from removal data. *Biometrics*, 34:621–630. 7

Chan, L., Silverman, B. W., and Vincent, K. (2021). Multiple systems estimation for sparse capture data: Inferential challenges when there are non overlapping lists. *Journal of the American Statistical Association*, 116:1297–1306. 5

Chao, A. (1987). Estimating the population size for capture-recapture data with unequal catchability. *Biometrics*, 43:783–791. 14, 15

Chao, A., Chu, W., and Chiu, H. H. (2000). Capture-recapture when time and behavioral response affect capture probabilities. *Biometrics*, 56:427–433. 10, 13

Chao, A. and Tsay, P. K. (1998). A sample coverage approach to multiple-system estimation with application to census undercount. *Journal of American Statistical Association*, 93:283–293. 5, 12, 13

Chao, A., Tsay, P. K., Lin, S. H., Shau, W. Y., and Chao, D. Y. (2001). The application of capture-recapture models to epidemiological data. *Statistics in Medicine*, 20:3123–3157. 11

Chatterjee, K. and Bhuyan, P. (2020). On the estimation of population size from a dependent triple-record system. *Journal of Royal Statistical Society, Series A*, 182:1487–1501. 5, 10, 13

Chatterjee, K. and Mukherjee, D. (2016a). An improved estimator of omission rate for census count: with particular reference to India. *Communication in Statistics: Theory and Methods*, 45:1047–1162. 7

Chatterjee, K. and Mukherjee, D. (2016b). On the estimation of homogeneous population size from a complex dual-record system. *Journal of Statistical Computation and Simulation*, 86:3562–3581. 10

Chatterjee, K. and Mukherjee, D. (2018). A new integrated likelihood for estimating population size in dependent dual-record system. *The Canadian Journal of Statistics*, 46:577–592. 14

Coull, B. A. and Agresti, A. (1999). The use of mixed logit models to reflect heterogeneity in capture-recapture studies. *Biometrics*, 55:294–301. 4

Darroch, J. N. (1981). The Mantel-Haenszel test and tests of marginal symmetry: Fixed-effects and mixed models for a categorical response. *International Statistical Review*, 49:285–307. 11

Darroch, J. N., Fienberg, S. E., Glonek, G. F. V., and Junker, B. W. (1993). A three-sample multiple-recapture approach to census population estimation with heterogeneous catchability. *Journal of the American Statistical Association*, 88:1137–1148. 11, 12, 13

Fienberg, S. E. (1972a). The analysis of incomplete multi-way contingency tables. *Biometrics*, 28:177–202. 9

Fienberg, S. E. (1972b). The multiple recapture census for closed populations and incomplete 2^k contingency tables. *Biometrika*, 59:591–603. 9, 13

Fienberg, S. E., Johnson, M. S., and W., J. B. (1999). Classical multilevel and bayesian approaches to population size estimation using multiple lists. *Journal of the Royal Statistical Society. Series A*, 162:383–405. 11, 12

Gold, S. J., Wibert, W. N., Bondartsova, V., Biroscak, B. J., and Post, L. A. (2015). A capture-recapture approach to estimation of refugee populations. *International Migration*, 53:3–25. 5

Gustafson, P. (2005). On model expansion, model contraction, identifiability and prior information: Two illustrative scenarios involving mismeasured variables. *Statistical Science*, 20:111–140. 5

Nour, E.-S. (1982). On the estimation of the total number of vital events with data from dual collection systems. *Journal of the Royal Statistical Society, Series A*, 145:106–116. 15

Otis, D. L., Burnham, K. P., White, G. C., and Anderson, D. R. (1978). Statistical inference from capture data on closed animal populations. *Wildlife Monographs: A Publication of Wildlife Society*, 62:3–135. 10

Rasch, G. (1961). On general laws and the meaning of measurement in psychology. *Proceedings of the Fourth Berkeley Symposium on Mathematical Statistics and Probability, ed. J. Neyman, Berkeley, CA: University of California Press*, 4.4:321–333. 10, 15

Sanathanan, L. (1972). Estimating the size of a multonomial population. *The Annals of Mathematical Statistics*, 43:142–152. 2

Stegelman, W. (1983). Expanding the Rasch model to a general model having more than one dimension. *Psychometrika*, 48:259–267. 11

Tanner, M. A. and Wong, W. H. (1987). The calculation of posterior distributions by data augmentation. *Journal of the American Statistical Association*, 82:528–540. 5

Wechsler, S., Izbicki, R., and Esteves, L. G. (2013). A bayesian look at nonidentifiability: A simple example. *The American Statistician*, 67:1537–2731. 5

Zaslavsky, A. M. and Wolfgang, G. S. (1993). Triple-system modeling of census, post-enumeration survey, and administrative-list data. *Journal of Business and Economic Statistics*, 11:279–288. 9
